# IMPDH inhibition induces DNA replication stress and ATR sensitivity in Merkel cell carcinoma

**DOI:** 10.1016/j.isci.2025.112567

**Published:** 2025-05-02

**Authors:** Julia L. Schnabel, Thomas C. Frost, Adam C. Wang, Varsha Ananthapadmanabhan, Satvik Gurram, Kara M. Soroko, Prafulla C. Gokhale, James A. DeCaprio

**Affiliations:** 1 Department of Medical Oncology, Dana-Farber Cancer Institute, Boston, MA, USA; 2 Program in Virology, Graduate School of Arts and Sciences, Harvard University, Cambridge, MA, USA; 3 Department of Medicine, Brigham and Women’s Hospital, Harvard Medical School, Boston, MA, USA; 4 Northeastern University, Dana-Farber/Harvard Cancer Center, CaNCURE, Cancer Nanomedicine Co-ops for Undergraduate Research Experience, Boston, MA, USA; 5 Experimental Therapeutics Core, Dana-Farber Cancer Institute, Boston, MA, USA

**Keywords:** Health sciences, Cell biology, Cancer systems biology

## Abstract

The rate-limiting isozyme of *de novo* guanosine biosynthesis, *IMPDH2*, was identified as an essential gene in Merkel cell carcinoma (MCC) but the consequences of its functional disruption were unclear. Inhibition of IMPDH2 led to reduced MCC cell viability, independent of functional p53 or Merkel cell polyomavirus status, but dependent on depletion of guanylate nucleotides. In contrast to other cancer models, inhibition of IMPDH2 in MCC led to rapid ablation of nascent DNA synthesis and the onset of replication stress without a significant effect on total or ribosomal RNA biosynthesis. Combining IMPDH inhibitors with ataxia telangiectasia mutated and Rad3-related (ATR) inhibitors significantly increased levels of replication stress *in vitro* and reduced tumor growth *in vivo*. These findings support replication stress as the dominant consequence of IMPDH2 inhibition in MCC and, when combined with ATR inhibition, indicate a potential therapeutic strategy.

## Highlights

• Merkel cell carcinoma (MCC) is highly susceptible to IMPDH inhibition
• IMPDH inhibition preferentially inhibits DNA synthesis relative to RNA synthesis in MCC
• IMPDH inhibition induces replication stress leading to p53 activation in MCC
• Combination of IMPDH and ATR inhibition leads to replication catastrophe

## Graphical abstract

**Figure:**
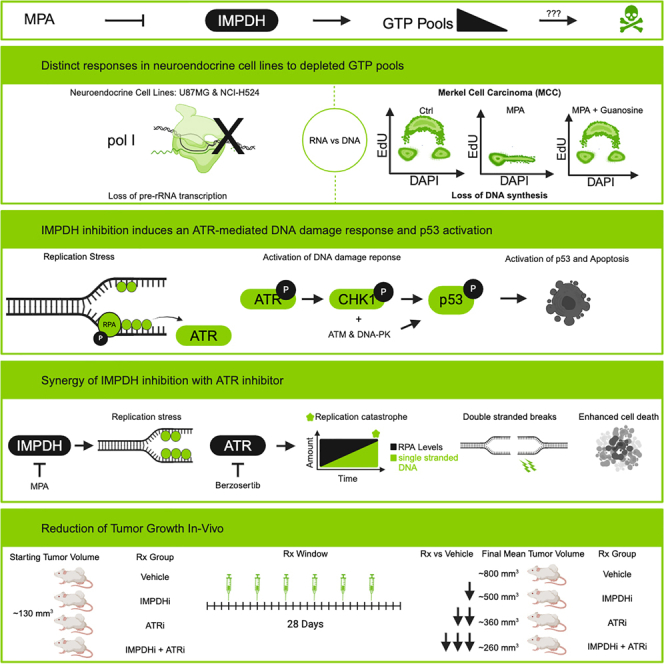

## Introduction

Merkel cell carcinoma (MCC) is an aggressive neuroendocrine carcinoma of the skin with two etiologies determined by the presence or absence of Merkel cell polyomavirus (MCPyV).^1^^,^^2^ Virus-negative MCC (MCCN) has a high tumor mutational burden due to extensive UV exposure and usually contains inactivating mutations in the *RB1* and *TP53* tumor suppressor genes.^3^^,^^4^ By contrast, polyomavirus-positive MCC (MCCP) contains clonally integrated copies of MCPyV DNA that constitutively express the viral small T antigen (ST) and a truncated form of large T antigen (LT). Although MCCP tumors typically contain wild type *RB1* and *TP53*, the viral T antigens can functionally suppress their activity. LT binds and inactivates the Rb protein to overcome the G1/S cell cycle checkpoint.^5^ ST forms a transcriptional activator complex with MYCL and the EP400/TIP60/NuA4 (from here on Tip60) chromatin modifying complex to drive expression of the E3 ubiquitin ligase MDM2 that targets p53 for ubiquitination and degradation by the proteasome.^6^^,^^7^ In response to cellular stress, p53 levels can increase, overcoming ST-mediated suppression and leading to apoptotic cell death.^6^^,^^8^^,^^9^^,^^10^

Despite the distinct genetic differences between MCCN and MCCP, clinical treatment of MCC is not stratified based upon viral subtype. Local disease is surgically excised followed by adjuvant radiotherapy while metastatic MCC has been historically treated with chemotherapy albeit with the rapid emergence of resistance.^1^^,^^11^^,^^12^^,^^13^^,^^14^ Recently, the approval of anti-PD-1/PD-L1 immune checkpoint inhibition (ICI) antibodies for the treatment of advanced and metastatic MCC has improved patient outcomes for patients who are eligible for treatment.^13^^,^^15^^,^^16^ However, approximately 50% of patients do not respond to ICI,^15^^,^^16^ and many who do respond become resistant, highlighting an urgent unmet need for additional therapies.

In addition to *MDM2*, the ST-MYCL-Tip60 complex drives expression of several genes that contribute to MCC oncogenesis. Integrated ChIP-seq and RNA-seq data identified inosine monophosphate dehydrogenase 2 (IMPDH2) as a specific downstream target of the ST-MYCL-Tip60 complex in MCCP.^7^ Furthermore, a genome-wide CRISPR-Cas9 knockout screen in the MCCP cell line MKL-1 identified *IMPDH2* as an essential gene. Treatment of MCC cell lines with the IMPDH inhibitor mycophenolate mofetil (MMF) treatment significantly reduces viability and inhibits tumor growth *in vivo*, but how MMF reduces cellular viability in MCC is not known.^17^ Given the significance of IMPDH2 in MCC biology and the clinical relevance of MMF, we sought to investigate the mechanism of IMPDH2 inhibition on cellular cytotoxicity in MCC.

IMPDH1 and IMPDH2 are the rate-limiting isozymes for *de novo* guanosine monophosphate (GMP) biosynthesis that convert inosine monophosphate (IMP) into xanthosine monophosphate (XMP) to generate GMP (Figure S1A).^18^ Newly synthesized guanylate nucleotides are used for nascent DNA and RNA synthesis, GTP-based signaling, and other functions.^18^ Although IMPDH1 and IMPDH2 share 84% amino acid identity and have nearly identical kinetic properties *in vitro*, IMPDH2 tends to be specifically upregulated in cancer.^18^ Inhibitors of IMPDH suppress both isozymes and IMPDH2 specific inhibitors are not available clinically.^19^ As such, IMPDH inhibition has been shown to deplete guanylate nucleotide levels and to induce cellular stress marked by the activation of p53.^20^^,^^21^^,^^22^^,^^23^

While IMPDH inhibition reduces cellular viability, the mechanism can vary among cancer types. One study observed prolonged S-phase and the accumulation of DNA damage, suggesting that IMPDH inhibition limits deoxynucleotide triphosphates (dNTPs) and DNA synthesis.^23^ However, other studies observed reduced levels of nascent pre-ribosomal RNA synthesis, indicating limited nucleotide triphosphates (NTPs).^20^^,^^21^ However, most studies did not assess the impact of IMPDH inhibition on DNA and RNA synthesis concurrently and it remains unclear what effects contribute to cellular cytotoxicity. Notably, metabolic analysis of normal and transformed cell lines showed baseline concentrations of GTP were substantially higher (65.7-fold) than dGTP,^24^ supporting the notion that DNA synthesis may be limiting in response to suppression of *de novo* guanylate nucleotide biosynthesis.

Depletion of dNTPs induces DNA replication stress (RS) in a variety of cancers^25^ and is the basis for many chemotherapeutics.^26^ In response to RS, ataxia telangiectasia and Rad3-related (ATR) is recruited to single-stranded DNA (ssDNA) by interacting with replication protein A (RPA), leading to activation of checkpoint kinase 1 (CHK1) and the intra-S checkpoint.^27^ The ATR-CHK1 intra-S phase checkpoint prevents mitotic entry prior to the completion of DNA replication and inhibits dormant origin firing to preserve genomic integrity.^27^^,^^28^ Inhibition of ATR in the presence of ongoing RS was shown to induce the widespread generation of ssDNA with subsequent recruitment of RPA through unchecked dormant origin firing.^29^ At a critical point, the amount of ssDNA generated can exceed global RPA levels resulting in replication exhaustion (RE). Exposed, unprotected ssDNA is highly susceptible to severe DNA damage and can lead to an end-stage event termed replication catastrophe (RC).^29^ Inhibition of ATR was found to enhance the efficacy of several RS-inducing chemotherapeutic agents.^30^^,^^31^^,^^32^ There is recent interest in MCC and other cancers for combining ATR inhibitors with immunotherapy to increase responses.^33^ MCC is highly sensitive to DNA damage,^9^^,^^34^ and the effects of IMPDH and ATR inhibitors have not been assessed. Here, we demonstrate that IMPDH inhibitors can induce RS in MCC cells, and when combined with ATR inhibitors, can lead to RC, offering a potent therapeutic strategy. This work not only advances our understanding of MCC biology but also opens new avenues for targeted treatments that could improve outcomes for patients with this challenging cancer.

## Results

### MCC cell lines are sensitive to IMPDH inhibition

**Figure 1:**
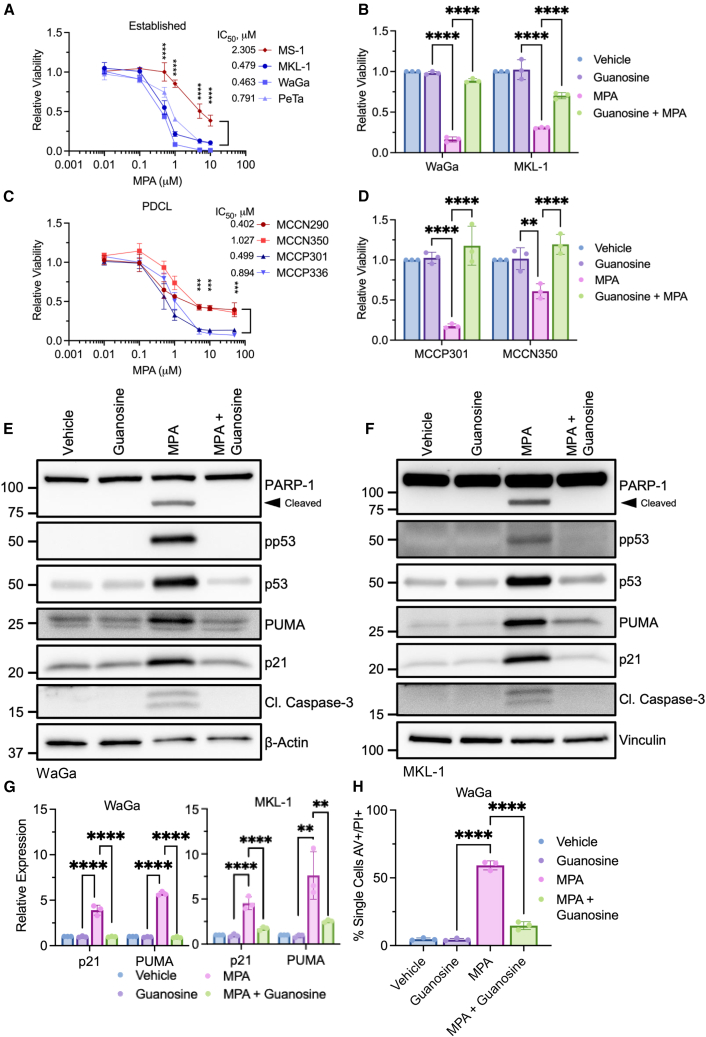
MCC cell lines are highly sensitive to IMPDH inhibition (A) CellTiter-Glo viability assay of established MCCP cell lines treated with increasing dosages of MPA for 3 days. Line color identifies p53-wild type (blue) and p53-mutant (red) cell lines. Statistics represent lowest significance (highest *p*) of any p53-wild type versus mutant comparisons at the specified dose. *N* = 3; mean ± SD; two-way ordinary ANOVA corrected for multiple comparisons via Tukey *post hoc* test; ∗*p* < 0.05, ∗∗*p* < 0.01, ∗∗∗*p* < 0.001, ∗∗∗∗*p* < 0.0001. The IC_50_ was calculated for each cell line. (B) CellTiter-Glo viability assay of WaGa and MKL-1 cell lines treated concurrently with MPA (1 μM) and guanosine (10 μM) for 3 days. *N* = 3; mean ± SD; two-way ordinary ANOVA corrected for multiple comparisons via Tukey *post hoc* test; ∗*p* < 0.05, ∗∗*p* < 0.01, ∗∗∗*p* < 0.001, ∗∗∗∗*p* < 0.0001. (C) CellTiter-Glo viability assay of PDCLs treated as in (A), with an additional dose of MPA (50 μM). Line coloring and statistical test are identical to (A); *N* = 3; mean ± SD. The IC_50_ was calculated for each cell line. (D) CellTiter-Glo viability assay of PDCLs treated concurrently with MPA (5 μM) and guanosine (1 μM) for 3 days. *N* = 3; mean ± SD; statistical tests are identical to (B). (E) Immunoblot of WaGa cells treated with MPA (1 μM) and guanosine (10 μM) for 24 h. Representative of 3 independent experiments. (F) Immunoblot of MKL-1 cells treated with MPA (1 μM) and guanosine (10 μM) for 3 days. Representative of 3 independent experiments. For panels E and F, total p53 is a reblot of the pp53 blot after stripping (see method details) and p21 is a reblot of PUMA. (G) RT-qPCR analysis of p53-dependent gene activation in WaGa and MKL-1 cells treated with MPA (1 μM) and guanosine (10 μM) for 1 or 3 days, respectively. Genes were normalized to the geometric mean of β-actin and β-2-microglobulin via the ΔΔCt method. *N* = 3; mean ± SD; one-way ordinary ANOVA corrected for multiple comparisons via Tukey *post hoc* test; ∗*p* < 0.05, ∗∗*p* < 0.01, ∗∗∗*p* < 0.001, ∗∗∗∗*p* < 0.0001. (H) AV/PI staining of WaGa cells treated with MPA (1 μM) and guanosine (10 μM) for 2 days. *N* = 3; mean ± SD; statistical tests are identical to (G). See also Figures S1G and S1H.

To assess the sensitivity of MCC to IMPDH inhibition, a panel of established MCCP cell lines were treated with the IMPDH inhibitor mycophenolic acid (MPA) and assessed for viability by ATP quantification (Figure 1A). Increasing dosages of MPA decreased viability in all cell lines tested. Notably, MKL-1, WaGa, and PeTa cell lines were significantly more sensitive than MS-1. MS-1 cells contain a mutation in *TP53* rendering the p53 protein non-functional in contrast to the other MCCP lines with wildtype *TP53*.^6^^,^^10^ Sensitivity to MPA treatment was recapitulated with direct measurement of viable cell counts (WaGa: Figure S1B). MKL-1 and WaGa cells were highly sensitive to additional IMPDH inhibitors including mycophenolate mofetil (MMF, CellCept), a prodrug formulation of MPA, and AVN-944 (VX-944) (WaGa & MKL-1: Figure S1C).

We assessed the effect of IMPDH inhibition relative nucleotide abundance in WaGa cells by liquid chromatography-mass spectrometry (LC-MS) (Figure S1D). Within 4 h of MPA treatment, a marked reduction (∼4-fold) in GMP nucleotides was observed and was sustained for at least 8 h. A substantial increase (∼10-fold) in levels of IMP, the specific nucleotide substrate of IMPDH1/2, was observed across all time points studied and as early as 2 h after MPA treatment (Figure S1D). Importantly, levels of AMP, the alternate nucleotide product of IMP conversion initiated by adenylosuccinate synthase, were unaffected over the course of the experiment (Figure S1D). Cell viability in WaGa and MKL-1 cell lines was rescued in a dose-dependent manner by cotreatment of MPA with guanosine (Figures 1B and S1E).

We assessed sensitivity to IMPDH inhibition in a panel of newly generated MCC patient-derived cell lines (PDCLs) including both MCCP cell lines (MCCP301 and MCCP336) with wildtype *TP53* and MCCN cell lines with mutated *TP53* (MCCN290 and MCCN350).^35^ Treatment with MPA decreased viability in a dose-dependent manner in all PDCLs, independent of viral and *TP53* status although PDCLs with wildtype *TP53* reached significantly higher maximal inhibition (Figure 1C). Sensitivity of the MCC PDCLs (MCCP301 and MCCN350) to IMPDH inhibition was rescued with guanosine co-treatment (Figures 1D and S1F).

Given the increased levels of cytotoxicity observed in MCC cells containing wildtype *TP53* when treated with MPA, we assessed for p53 activation in WaGa and MKL-1 by immunoblot (Figures 1E and 1F). Treatment with MPA for 24 h induced p53 phosphorylation (Ser15; pp53) coinciding with the accumulation of total p53 protein. In addition, levels of downstream p53 targets including p21 (*CDKN1A*) and PUMA (*BBC3*) protein and mRNA (Figures 1E–1G) increased in WaGa and MKL-1 cells, indicative of activation of the p53 transcriptional program in response to IMPDH inhibition.

To assess for apoptosis induced by IMPDH inhibition, we performed annexin-V (AV) and propidium iodide (PI) staining in WaGa cells (Figures 1H and S1G, S1H). Strikingly, within two days of treatment, a significant population of apoptotic (∼11%, AV+/PI-) and dead cells (∼60%, AV+/PI+) was observed. Levels of apoptosis were significantly reduced and viability increased with guanosine co-treatment. Collectively, these findings indicate that IMPDH inhibition can lead to p53 activation and trigger apoptotic cell death.

### Sensitivity to IMPDH inhibition is enhanced by p53 but is not required

**Figure 2:**
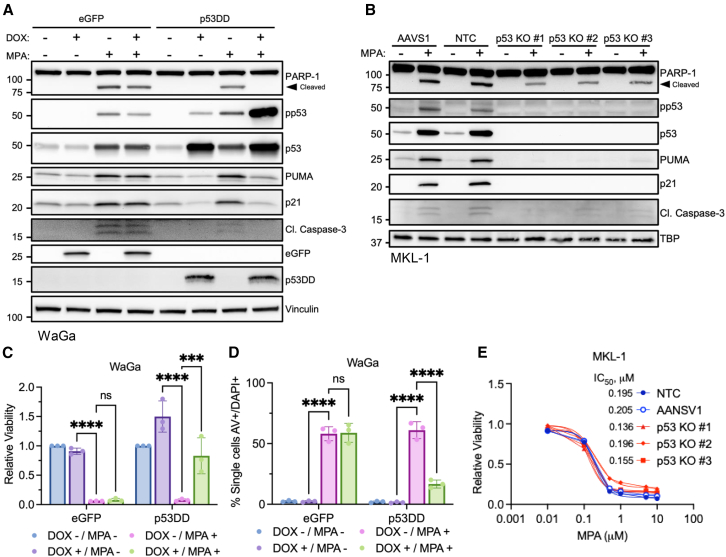
Functional p53 enhances IMPDH inhibition-induced cytotoxicity but is not required for cytotoxicity in MCC (A) Immunoblot of WaGa cells with inducible dominant negative p53 (p53DD) or eGFP pretreated with DOX (1 μg/mL) for 24 h followed by MPA (1 μM) for an additional 24 h. Representative of 3 independent experiments. (B) Immunoblot of MKL-1 clonal p53 knockout (KO) or control (AANSV1 and NTC) cells treated with MPA (1 μM) for 3 days. Representative of 3 independent experiments. For panels A and B, total p53 is a reblot of pp53 and p21 is a reblot of PUMA. (C) CellTiter-Glo viability assay of WaGa cells pretreated with DOX (1 μg/mL) for 24 h followed by MPA (1 μM) for 3 days. *N* = 3; mean ± SD; two-way ordinary ANOVA corrected for multiple comparisons via Tukey *post hoc* test; ∗*p* < 0.05, ∗∗*p* < 0.01, ∗∗∗*p* < 0.001, ∗∗∗∗*p* < 0.0001. (D) AV/DAPI staining of WaGa cells treated as in (C) for 2 days. *N* = 3; mean ± SD; statistical tests are identical to (C). See also Figures S2B and S2C. (E) CellTiter-Glo viability of clonal MKL-1 control and p53 KO cell lines treated with increasing dosages of MPA for 3 days. *N* = 3; mean ± SD. Statistical tests are identical to (C). No statistical significance was identified. The IC_50_ was calculated for each cell line.

Given the strong activation of p53 in MKL-1 and WaGa cell lines in response to IMPDH inhibition, we sought to determine if wild type p53 activity was required for MPA-induced cytotoxicity. WaGa cells were transduced with doxycycline-inducible vectors expressing either dominant negative p53DD^36^ or enhanced green fluorescent protein (eGFP) as a control.^9^ Disruption of p53 activity in p53DD expressing cells compared to the eGFP control was confirmed by increased resistance to the MDM2 inhibitor Nutlin-3a (Figure S2A). As expected, induction of p53DD led to increased levels of endogenous p53 due to tetramer-mediated stabilization (Figure 2A).^37^^,^^38^^,^^39^ Expression of p53DD dampened the p53 response in cells with the reduced levels of p21, PUMA, and cleaved PARP-1 and Caspase-3 in response to MPA treatment. In addition, we observed reduced levels of p21, PUMA, cleaved PARP-1 and Caspase-3 in several clones of *TP53* knockout (p53 KO) MKL-1 cells^8^ treated with MPA relative to the AAVS1 safe harbor or non-targeting control (NTC) cells (Figure 2B).

Expression of p53DD partially rescued cell viability and significantly reduced the number of apoptotic cells when compared to eGFP or the uninduced controls in response to MPA treatment (Figures 2C, 2D and S2B, S2C). Strikingly, MPA treatment had minimal effect on sensitivity to IMPDH inhibition in MKL-1 p53 KO cells compared to controls (Figure 2E).

### IMPDH inhibition rapidly abolishes dNTP incorporation into nascent DNA

Loss of GMP nucleotides has been reported to limit nascent RNA^20^^,^^21^ and DNA^23^ synthesis, but the relative contributions of these events have not been explored simultaneously. Previous reports show the consequences of IMPDH inhibition on RNA synthesis impair ribosome biogenesis through the loss of RNA polymerase I-dependent transcription of pre-ribosomal RNA for the 18S rRNA transcript.^20^^,^^21^ Given that multiple pathways rely on the generation of GMP nucleotides through *de novo* purine biosynthesis, we sought to investigate what pathways were affected by IMPDH inhibition in MCC.

**Figure 3:**
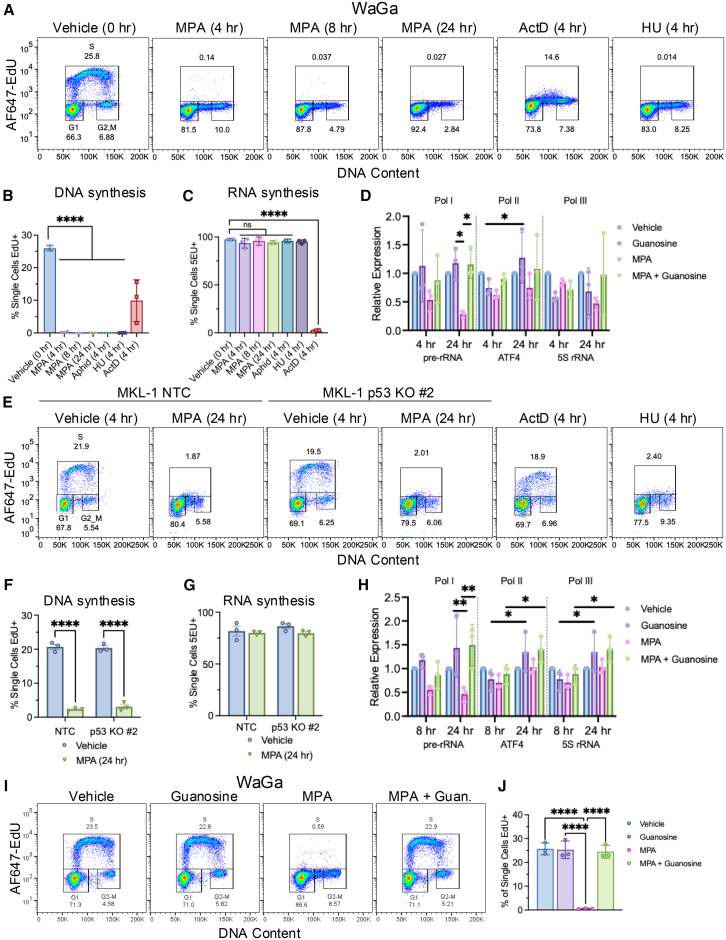
IMPDH inhibition rapidly abolishes dNTP incorporation into nascent DNA in MCC cell lines (A) Flow cytometry analysis of WaGa cells treated with MPA (1 μM), ActD (1 μM), and HU (1 mM) for times indicated and pulsed with EdU (10 μM) in the final hour. Images generated in FlowJo. Representative of 3 independent experiments. (B) Quantification of single cells positive for EdU from WaGa cells treated with MPA (1 μM), ActD (1 μM), aphidicolin (10 μM), and HU (1 mM) for up to 24 h, pulsed with EdU (10 μM) in the final hour. *N* = 3; mean ± SD; one-way ordinary ANOVA corrected for multiple comparisons via Tukey *post hoc* test; ∗*p* < 0.05, ∗∗*p* < 0.01, ∗∗∗*p* < 0.001, ∗∗∗∗*p* < 0.0001. (C) Quantification of single cells positive for 5EU from WaGa cells treated as in (B) but pulsed with 5EU (1 mM) in the final hour. *N* = 3; mean ± SD; statistical tests are identical to (B). See also Figures S3A and S3B. (D) RT-qPCR analysis of the effect of MPA treatment at 4 h and 24 h on RNA polymerase I (pre-rRNA), II (ATF4), and III (5S rRNA) gene targets in WaGa cells. *N* = 3; mean ± SD; two-way ordinary ANOVA corrected for multiple comparisons via Tukey *post hoc* test; ∗*p* < 0.05, ∗∗*p* < 0.01, ∗∗∗*p* < 0.001, ∗∗∗∗*p* < 0.0001. (E) Flow cytometry analysis of MKL-1 control cells versus MKL-1 p53 KO #2 cells treated with MPA (1 μM), ActD (1 μM), and HU (1 mM) for indicated times, pulsed with EdU (10 μM) in the final hour. Images generated in FlowJo. Representative of 3 independent experiments. (F) Quantification of single cells positive for EdU from flow cytometric analysis of MKL-1 control cells versus MKL-1 p53 KO #2 cells treated with MPA (1 μM) for times indicated and pulsed with EdU (10 μM) in the final hour. *N* = 3; mean ± SD; *N* = 3; mean ± SD; two-way ordinary ANOVA corrected for multiple comparisons via Tukey *post hoc* test; ∗*p* < 0.05, ∗∗*p* < 0.01, ∗∗∗*p* < 0.001, ∗∗∗∗*p* < 0.0001. (G) Quantification of single cells positive for 5EU from flow cytometric analysis of MKL-1 control cells versus MKL-1 p53 KO #2 cells treated with MPA (1 μM), ActD (1 μM), and HU (1 mM) for up to 24 h and pulsed with 5EU (1 mM) in the final hour. *N* = 3; mean ± SD; statistical tests are identical to (F). See also Figures S3C and S3D. (H) RT-qPCR analysis of the effect of MPA treatment at 8 and 24 h on RNA polymerase I, II, and III gene targets in MKL-1 cells. *N* = 3; mean ± SD; statistical tests are identical to (D). (I) Flow cytometry analysis of WaGa cells treated concurrently with MPA (1 μM) and guanosine (10 μM) for 4 h, pulsed with EdU (10 μM) in the final hour. Images generated in FlowJo. Representative of 3 independent experiments. (J) Quantification of single cells positive for EdU from (I). *N* = 3; mean ± SD; statistical tests are identical to (B).

To assess levels of total DNA and RNA synthesis, WaGa cells were treated with MPA and 5-ethynyl-2′-deoxyuridine (EdU) (Figure 3A) to label newly synthesized DNA or 5-ethynyl uridine (5EU) (Figure S3A), to label nascent RNA and measured by flow cytometry. Treatment with hydroxyurea (HU) for 4 h completely ablated DNA synthesis (Figures 3A and 3B), while not significantly perturbing the percentage of nascent RNA-labeled cells (Figures 3C and S3A) or the mean fluorescent intensity (MFI) of NTP incorporation (Figure S3B). Actinomycin D (ActD) completely blocked nascent RNA production and partially suppressed DNA synthesis, though not to the extent of either aphidicolin or HU (Figures 3B, 3C and S3A, S3B).

Strikingly, MPA ablated nascent dNTP incorporation occurred within 4 h of treatment and this effect was sustained for 8 and 24 h in WaGa cells (Figures 3A and 3B). Notably, this timeline coincided with the maximal depletion of GMP nucleotides as detected by LC-MS (Figure S1D). By contrast, MPA had no significant effect on the proportion of cells positive for nascent RNA synthesis (Figures 3C and S3A) or the mean fluorescence intensity (MFI) of cells with incorporated 5EU (Figure S3B).

We assessed the effect of IMPDH inhibition on the activity of RNA polymerase I (Pol I), RNA polymerase II (Pol II), and RNA polymerase III (Pol III) in WaGa cells by measuring the relative expression of a representative target gene for each RNA polymerase using RT-qPCR as previously described.^20^ The candidate genes include pre-rRNA for Pol I, ATF4 for Pol II*,* and 5S rRNA for Pol III. Following 4 h of MPA treatment in WaGa cells, at which point DNA synthesis was completely ablated (Figure 3B), there was a non-significant reduction in the Pol I, II, and III dependent transcripts compared to vehicle, guanosine treatment, or MPA guanosine co-treatment (Figure 3D). By 24 h, pre-rRNA levels were significantly reduced with MPA treatment compared to guanosine treatment and MPA guanosine co-treatment indicating that MPA treatment was on target and could be rescued with guanosine treatment. There was no significant reduction in transcript levels for the Pol II and Pol III targets ATF4 and 5S rRNA with MPA treatment. There was a significant increase in ATF4 transcript levels over time with guanosine treatment (Figure 3D).

To determine if IMPDH inhibition had similar effects on DNA and RNA synthesis in MCC cells lacking p53, we treated the MKL-1 p53 KO cells with MPA for 24 h followed by EdU and 5EU labeling. At 24 h, we observed a complete loss of EdU+ labeled cells in MKL-1 control and p53 KO cells indicating shutdown of DNA synthesis (Figures 3E and 3F) and no difference in the 5EU + cell population indicating ongoing nascent RNA synthesis (Figures 3G and S3C). No significant difference was observed for the MFI of 5EU incorporation with MPA treatment, while there was a significant decrease observed in the MFI with ActD treatment compared to vehicle. (Figure S3D).

Similar to WaGa cells, we did not observe a significant decrease in pre-rRNA levels in MKL-1 cells at 8 or 24 h with MPA treatment compared to the vehicle (Figure 3H). This supports that the loss of DNA synthesis was preferentially decreased compared to pre-rRNA synthesis in MCC. We observed that guanosine supplementation and guanosine rescue of MPA led to significantly increased levels of ATF4 and 5s rRNA at 8 h and 24 h (Figure 3H).

To confirm the on-target effects of MPA on nascent DNA synthesis, we co-treated WaGa cells with MPA and guanosine followed by EdU labeling (Figures 3I and 3J). MPA treatment rapidly reduced dNTP incorporation, an effect that was completely rescued by the supplementation of guanosine. Loss of dNTP incorporation upon MPA treatment was also observed in MCCP301, and this effect was rescued by guanosine supplementation (Figure S3E). These results strongly implicate the suppression of nascent DNA synthesis as the dominant effect of IMPDH inhibition in MCC.

### Preferential suppression of DNA synthesis by IMPDH inhibition is not conserved in other cancer cell lines

**Figure 4:**
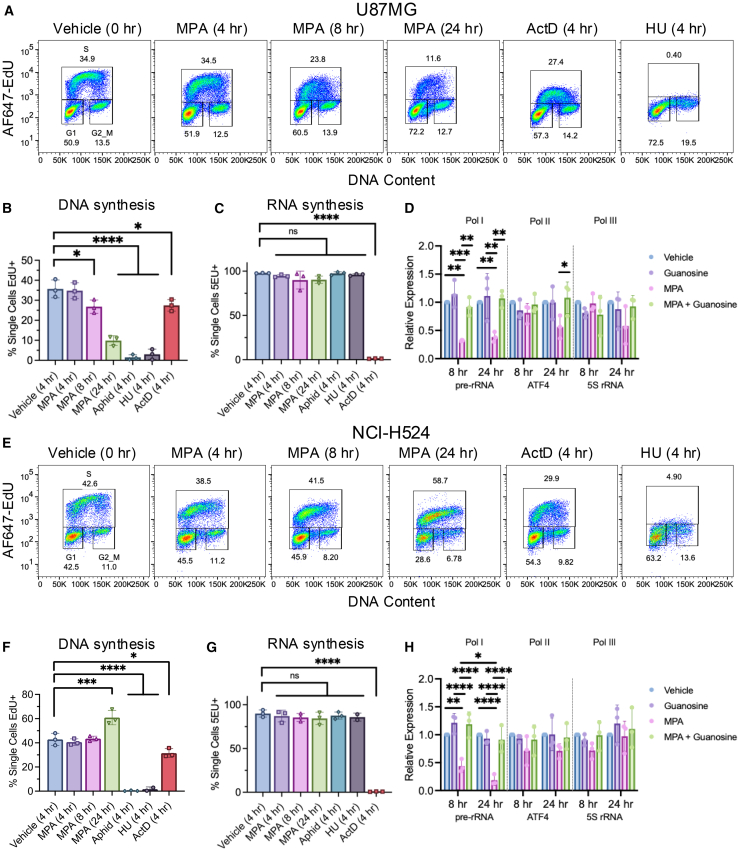
Preferential loss of DNA synthesis from IMPDH inhibition is not conserved in other relevant cancer cell lines (A–D) Representing data for U87MG (*TP53* wild type) glioblastoma cell lines. (A) Flow cytometry analysis of U87MG cells treated with MPA (1 μM), ActD (1 μM), and HU (1 mM) for indicated times, pulsed with EdU (10 μM) in the final hour. Images generated in FlowJo. Representative of 3 independent experiments. (B) Quantification of single cells positive for EdU from U87MG cells treated with MPA (1 μM), ActD (1 μM), aphidicolin (10 μM), and HU (1 mM) for up to 24 h, pulsed with EdU (10 μM) in the final hour. *N* = 3; mean ± SD; one-way ordinary ANOVA corrected for multiple comparisons via Tukey *post hoc* test; ∗*p* < 0.05, ∗∗*p* < 0.01, ∗∗∗*p* < 0.001, ∗∗∗∗*p* < 0.0001. (C) Quantification of single cells positive for 5EU from U87MG cells treated as in (B) and pulsed with 5EU (0.5 mM) in the final hour. *N* = 3; mean ± SD; statistical tests are identical to (B). See also Figures S4C and S4D. (D) RT-qPCR analysis of the effect of MPA treatment at 8 h and 24 h on RNA polymerase I (pre-rRNA), II (ATF4), and III (5S rRNA) gene targets in U87MG cells. *N* = 3; mean ± SD; two-way ordinary ANOVA corrected for multiple comparisons via Tukey *post hoc* test; ∗*p* < 0.05, ∗∗*p* < 0.01, ∗∗∗*p* < 0.001, ∗∗∗∗*p* < 0.0001. (E-H) Representing data for the NCI-H524 (*TP53* mutant) small cell lung cancer cell line. (E) Flow cytometry analysis of NCI-H524 cells treated with MPA (1 μM), ActD (1 μM), and HU (1 mM) for indicated times, pulsed with EdU (10 μM) in the final hour. Representative of 3 independent experiments. (F) Quantification of single cells positive for EdU from NCI-H524 cells treated with MPA (1 μM), ActD (1 μM), aphidicolin (10 μM), and HU (1 mM) for up to 24 h, pulsed with EdU (10 μM) in the final hour. *N* = 3; mean ± SD; statistical tests are identical to (B). (G) Quantification of single cells positive for 5EU from NCI-H524 cells treated as in (F) but pulsed with 5EU (0.5 mM) in the final hour. *N* = 3; mean ± SD; statistical tests are identical to (B). See also Figures S4E and S4F. (H) RT-qPCR analysis of the effect of MPA treatment at 8 and 24 h on RNA polymerase I, II, and III gene targets in NCI-H524 cells. *N* = 3; mean ± SD; statistical tests are identical to (D).

We compared the effects of IMPDH inhibition on DNA and RNA synthesis in the cell lines U87MG (glioblastoma, wild type *TP53*) and NCI-H524 (small cell lung cancer, mutant *TP53*) used in prior studies.^20^^,^^21^ Increasing doses of MPA decreased cell viability at similar levels in both cell lines (U87MG: Figure S4A, NCI-H524; Figure S4B). In U87MG cells, although DNA synthesis was not reduced after 4 h of MPA treatment, it was significantly reduced after 8 h (*p* < 0.05) and 24 h (*p* < 0.0001) (Figures 4A and 4B), while total RNA synthesis was unaffected (Figures 4C and S4C). As expected, HU and aphidicolin abolished nascent DNA synthesis and did not affect total RNA synthesis (Figures 4A–4C), while ActD completely abolished RNA synthesis and partially reduced DNA synthesis (Figures 4A–4C and S4C). Of note, there was a significant decrease in the MFI of 5EU incorporation with MPA treatment at 24 h compared to control (*p* < 0.05) (Figure S4D).

Consistent with an earlier report, we observed a significant reduction (*p* < 0.01) in pre-rRNA levels in U87MG cells with MPA (1 μM) treatment at 8 and 24 h that was rescued with guanosine co-treatment (Figure 4D).^21^ We did not find significant differences in ATF4 and 5s rRNA levels with MPA treatment, also consistent with the previous report.^21^ Similarly, in NCI-H524 cells we observed IMPDH inhibition significantly impaired pre-rRNA synthesis while Pol II and Pol IIl transcripts were unaffected and there were no significant changes in total RNA synthesis with MPA treatment (Figures 4G, 4H, S4E, S4F). Notably, we observed a significant increase in DNA synthesis at 24 h with MPA (1 μM) treatment (Figures 4E and 4F). We observed that pre-rRNA levels were significantly reduced in the MPA treated cells compared to vehicle starting at 8 h (*p* < 0.01) becoming more significantly reduced at 24 h (*p* < 0.0001) (Figure 4H). These results indicate that IMPDH inhibition preferentially affects Pol I dependent transcription of pre-rRNA and does not impact DNA synthesis in NCI-H524 cells consistent with the results reported in Huang et al.^20^

### IMPDH inhibition induces replication stress in MCC

**Figure 5:**
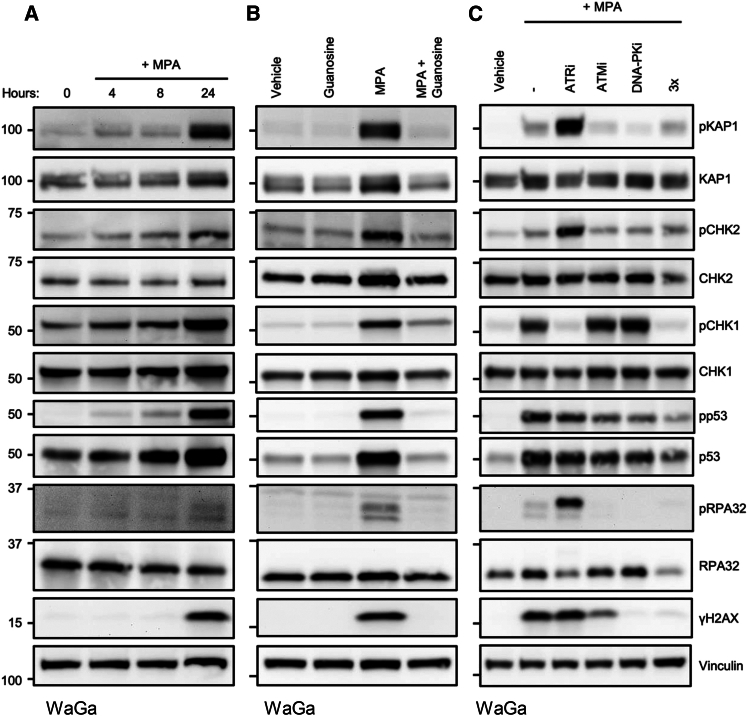
Activation of p53 by IMPDH inhibition is dependent on RS (A) Immunoblot of WaGa cells treated with MPA (1 μM) for indicated times. Representative of 3 independent experiments. (B) Immunoblot of WaGa cells treated with MPA (1 μM) and guanosine (10 μM) for 24 h. Representative of 3 independent experiments. (C) Immunoblot of WaGa cells treated for 24 h with MPA (1 μM) concurrent with inhibitors for ATR (250 nM; berzosertib), ATM (10 μM; KU55933), DNA-PK (10 μM; AZD7648) or with all three inhibitors combined (3x). Representative of 3 independent experiments. For panels A-C, the total KAP1 blot is a reblot of the pKAP1 blot, the total CHK1 blot is a reblot of pCHK1, and the total CHK2 blot is a reblot of pCHK2.

Given the rapid ablation of nascent DNA synthesis following IMPDH inhibition in MCC, we suspected that this led to RS. Indeed, treatment with MPA has been previously reported to induce specific phosphorylation of CHK1, RPA32, and H2AX.^23^ To determine if IMPDH inhibition induced RS in MCC, we treated WaGa cells with MPA and assessed the response by immunoblot (Figure 5A). We observed activation of RS markers including phosphorylation of KAP1 (Ser824; pKAP1),^40^ CHK2 (Thr68; pCHK2),^41^ CHK1 (Ser317; pCHK1),^41^ and RPA32 (Ser8; pRPA32)^42^ as early as 4 h of MPA treatment with maximal response by 24 h. The presence of double-stranded DNA breaks (DSBs) as marked by H2AX Ser139 phosphorylation (γH2AX) was detected at 24 h (Figure 5A). Co-treatment with MPA and guanosine reduced the levels of the RS markers to baseline (Figure 5B).

To determine if sensitivity to IMPDH inhibition depended on the stimulation of specific DNA damage kinases, we treated WaGa cells with MPA and inhibitors of ATR (berzosertib), ATM (KU55933), DNA-PK (AZD7648), or the combination of all three inhibitors (Figure 5C). Notably, ATR inhibition combined with MPA treatment markedly increased the levels of pKAP1, pCHK2, and pRPA32 relative to MPA alone, an effect not observed with the other kinase inhibitors. As expected, ATR inhibition led to decreased levels of its direct target pCHK1. ATM inhibition led to lower levels of pRPA32 while DNA-PK inhibition led to lower levels of pKAP1, pRPA32, and γH2AX. In response to MPA and individual inhibition of ATR, ATM, or DNA-PK, the accumulation of total p53 and Ser15 phosphorylation of p53 was modestly decreased. However, when all three inhibitors were combined with MPA, activation of p53 was reduced. These results implicate RS as a major consequence of IMPDH inhibition that was enhanced by ATR inhibition.

### Dual-inhibition of IMPDH and ATR induces p53-independent replication catastrophe

**Figure 6:**
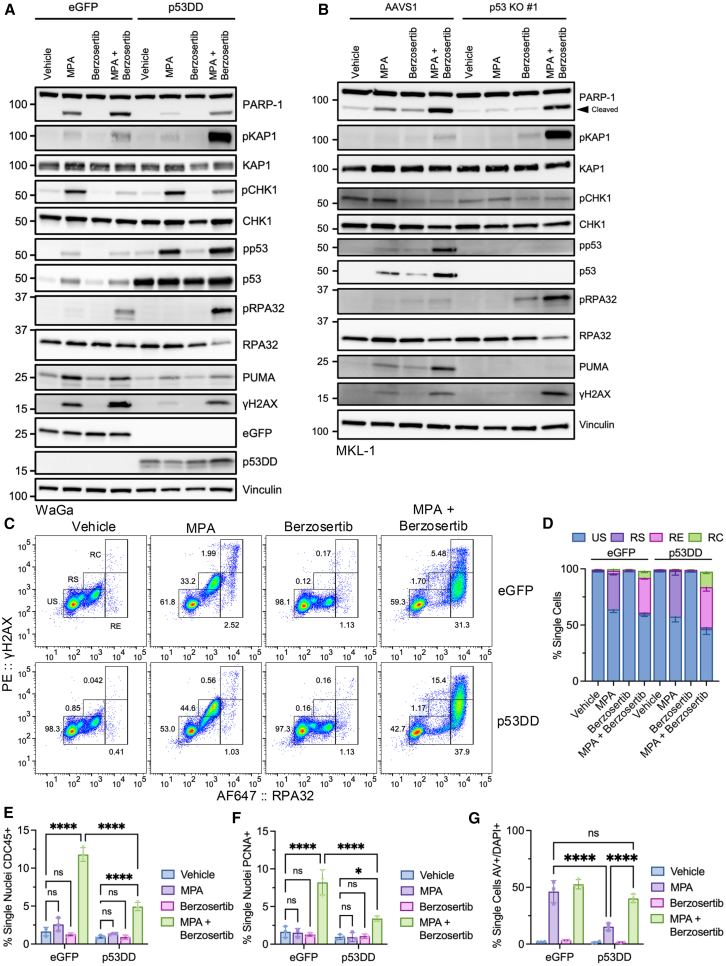
Dual inhibition of IMPDH and ATR induces p53-independent replication catastrophe (A) Immunoblot of WaGa cells pre-induced with DOX (1 μg/mL) for 24 h to express p53DD or eGFP followed by treatment with MPA (1 μM) and berzosertib (250 nM) for an additional 24 h. Representative of 3 independent experiments. (B) Immunoblot of MKL-1 p53 KO or control (AAVS1) cells treated with MPA (1 μM) and berzosertib (250 nM) for 3 days. Representative of 3 independent experiments. For panels A and B, the total p53 blot is a reblot of pp53, the total KAP1 blot is a reblot of the pKAP1 blot, the total CHK1 blot is a reblot of pCHK1, and the total RPA32 blot is a reblot of pRPA32. (C) Flow cytometry analysis of chromatin-associated γH2AX and RPA32 in WaGa cells pre-induced and treated as in (A). Gating strategy for each defined population is shown. Images generated in FlowJo. Representative of 3 independent experiments. (D) Quantification of populations from (C). *N* = 3; mean ± SD. (E) Quantification of chromatin-associated CDC45 from WaGa cells treated as in (A). *N* = 3; mean ± SD; two-way ordinary ANOVA corrected for multiple comparisons via Tukey *post hoc* test; ∗*p* < 0.05, ∗∗*p* < 0.01, ∗∗∗*p* < 0.001, ∗∗∗∗*p* < 0.0001. (F) Quantification of chromatin-associated PCNA from WaGa cells treated as in (A). *N* = 3; mean ± SD; statistical tests are identical to (E). (G) Quantification of WaGa single cells dual-positive for AV and DAPI prepared as in (A) but treated with inhibitors for 2 days. *N* = 3; mean ± SD; statistical tests are identical to (E).

Recent work has shown that dysregulation of the ATR-CHK1 checkpoint can generate extensive DSBs and induce cell death.^28^^,^^29^^,^^30^^,^^31^ Since IMPDH inhibition led to RS, we suspected that combining IMPDH with ATR inhibition could enhance cytotoxicity, even in cells without functional p53. We induced expression of p53DD or eGFP in WaGa cells, treated with MPA and the ATR inhibitor berzosertib, and assessed for markers of RS and apoptosis by immunoblot (Figure 6A). MPA treatment alone induced accumulation of apoptotic markers that were suppressed by p53DD induction. Treatment with berzosertib alone suppressed baseline levels of pCHK1 but was otherwise unremarkable compared to vehicle. Strikingly, co-treatment with MPA and berzosertib enhanced activation of RS and apoptotic markers compared to MPA alone, an effect independent of functional p53. Notably, induction of p53DD led to dramatically increased accumulation of RS markers pKAP1 and pRPA32 relative to eGFP upon dual-treatment. The increased sensitivity to RS in p53-suppressed conditions is consistent with previous reports that demonstrated enhanced sensitivity to ATR inhibitors with the loss of p53 activity.^43^ We observed similarly enhanced responses including pKAP1 and pRPA32 when combining MPA and berzosertib in MKL-1 p53 KO cells (Figure 6B).

To further characterize the mechanism of sensitivity to combined ATR and IMPDH inhibition, we performed chromatin flow cytometry to assess the presence of RS markers on DNA.^29^^,^^40^^,^^44^ WaGa cells were induced to express p53DD or eGFP and treated with MPA and berzosertib. Antibody staining for chromatin-associated RPA32 and γH2AX^29^ identified cellular populations undergoing RS (γH2AX+, RPA32+), replication exhaustion (RE; γH2AX+/−, RPA32-hi), and replication catastrophe (RC; γH2AX-hi, RPA32-hi) upon treatment with MPA and berzosertib (Figures 6C and 6D). Unstressed cells (US) lacked γH2AX but could be positive for RPA32 (γH2AX-, RPA32^+/−^). MPA treatment alone significantly increased the proportion of cells undergoing RS (30/40% for eGFP/p53DD, respectively) but did not reach the threshold for RE or RC, regardless of p53 status (Figures 6C and 6D). Berzosertib alone did not induce RS, RE, or RC. However, dual-inhibition of IMPDH and ATR led to a significantly increased proportion of cells undergoing RE (30–35%) and RC (5–15%) relative to single-treated and untreated cells, irrespective of p53 status. Remarkably, the MFI for RPA32 in dual-treated cells increased ∼7-fold (Figure S5A). Consistent with previous reports, only RPA32-hi cells were observed to undergo RC suggesting that cellular RPA32 levels were completely exhausted.^28^^,^^29^ We observed that the proportion of RPA32+ single cells significantly increased upon MPA or dual-treatment (Figure S5B). When we split the cell populations into 2N and 4N content we observed a significant increase in RPA32+ cells in the 2N population of MPA and dual-treatment compared to vehicle or berzosertib alone (Figures S5C and S5E). The presence of RPA32+ cells in the 2N population suggests DNA replication was initiated despite the lack of guanosine nucleotides. Taken together, these results indicate that combined inhibition of IMPDH and ATR led to significantly increased levels of chromatin bound RPA32 and γH2AX and the induction of RC independent of p53 status.

Inhibition of ATR can lead to increased dormant origin firing, resulting in the accumulation of unprotected ssDNA and subsequent induction of RS.^29^ We addressed this possibility by assessing levels of chromatin-associated CDC45 (Figures 6E and S5F) and PCNA (Figures 6F and S5F). Chromatin-association of CDC45 and PCNA have been used to assess origin firing and active DNA replication, respectively.^40^ Single-treatment of MPA or berzosertib did not affect levels of chromatin-associated CDC45 or PCNA compared to untreated cells. However, combined MPA and berzosertib induced significant accumulation of CDC45 and PCNA on chromatin. Cells with functional p53 (eGFP) had increased loading of CDC45 and PCNA relative to those with non-functional p53 (p53DD). This may have been due to a higher proportion of cells with non-functional p53 undergoing RC and thus accruing extensive DNA damage (Figures 6C and 6D). Thus, dual-treatment with ATR and IMPDH inhibitors results in dormant origin firing in the presence of a replicative block induced by MPA.

RC leads to the accumulation of extensive and irreparable DSBs and results in cell death.^28^^,^^29^ To determine if dual-treatment could enhance cytotoxicity, we assessed the degree of apoptosis by AV/DAPI (Figure 6G) and TUNEL (Figure S5D) staining in p53DD or eGFP-expressing WaGa cells treated with MPA and berzosertib. In both assays, MPA treatment alone significantly increased the proportion of dead cells (AV+/DAPI+ or TUNEL+), an effect that was significantly reduced by p53DD expression. Berzosertib treatment alone did not induce cell death in either assay. Strikingly, combination of ATR and IMPDH inhibition significantly increased cytotoxicity compared to MPA treatment alone in p53DD expressing cells.

### Combination of IMPDH and ATR inhibition controls tumor growth in MCC xenografts

**Figure 7:**
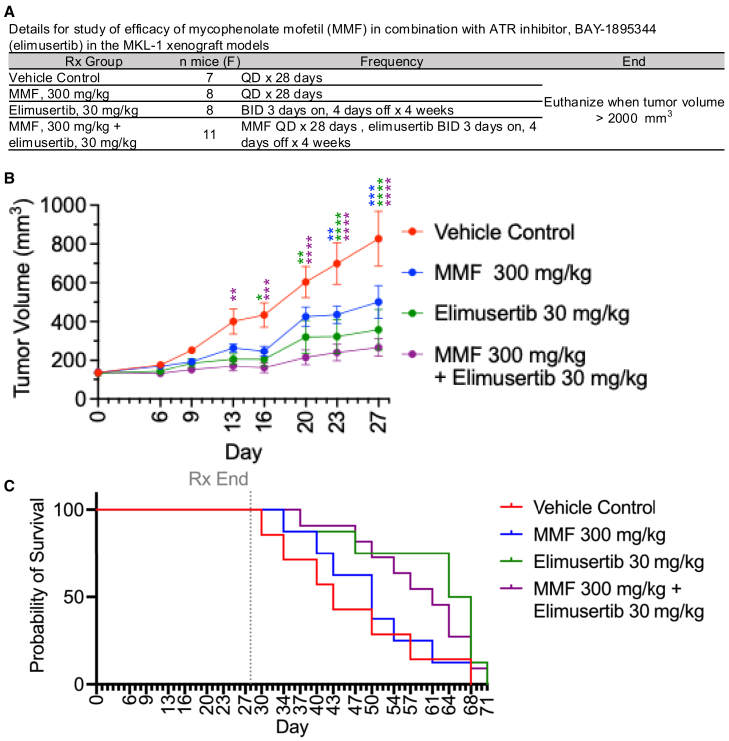
Combination treatment of IMPDH inhibitor, MMF, and ATR inhibitor, elimusertib, in MKL-1 xenograft models significantly reduces tumor growth (A) Treatment groups for MKL-1 xenograft mouse models. QD = treatment every day. BID = Twice a day. (B) Tumor volume measurements by treatment arm and duration of treatment. Mean ± SEM. Statistical analysis of treatments versus vehicle at each time point assessed through two-way ordinary ANOVA corrected for multiple comparisons via Tukey *post hoc* test, ∗*p* < 0.05, ∗∗*p* < 0.01, ∗∗∗*p* < 0.001, ∗∗∗∗*p* < 0.0001. Color of asterisk corresponds to the treatment group compared to the vehicle. See also Figure S6A. (C) Survival curves. Statistical analysis of survival distributions of treatment groups compared to vehicle group, Mantel-Cox with Bonferroni multiple comparing correction, ∗*p* < 0.0167. vehicle vs. MMF, *p* = 0.68; vehicle vs. elimusertib, *p* = 0.046; vehicle vs. combination, *p* = 0.092.

Given the effect of combining ATR and IMPDH on MCC cytotoxicity *in vitro*, we investigated whether this effect could be recapitulated *in vivo*. For this study, we used elimusertib, an orally available ATR inhibitor, instead of berzosertib, which requires intravenous administration, for ease of use and consistent dosing.^45^ MKL-1 xenografts were treated with vehicle, MMF, elimusertib, or combination MMF and elimusertib for 28 days (Figures 7A and S6A–S6D). By day 13 of treatment, the mean tumor volume of combination MMF and elimusertib was significantly reduced compared to the mean tumor volume of the vehicle. There was no significant difference in the mean tumor volume of single arm treatment of MMF or elimusertib compared to vehicle mean tumor volume at day 13 (Figure 7B). For the rest of the treatment window, combination treatment significantly reduced the mean tumor volume compared to vehicle. Elimusertib alone significantly reduced the mean tumor volume compared to the vehicle mean tumor volume by day 16 and MMF significantly reduced the mean tumor volume by day 23 compared to the vehicle. These results suggest combination treatment with ATR and IMPDH inhibitors outperforms single arm treatments for reducing tumor growth during the treatment window and supports a viable treatment strategy for controlling MCC *in vivo*.

Following treatment completion, we assessed the survival distributions of each treatment group (MMF, elimusertib, and combination treatment) compared to the vehicle group using individual log-rank tests (Mantel-Cox) where the event of interest was reaching a tumor volume of 2000 mm^3^ (Figure 7C). To account for multiple comparisons, we adjusted the significance level (*p* < 0.0167) using the Bonferroni method which divides the original significance level (*p* < 0.05) by the number of comparisons being made (*n* = 3) to reduce type-I errors. Using this method, we found the following *p*-values: MMF compared to control (*p* = 0.682), elimusertib compared to control (*p* = 0.046), and combination treatment compared to control (*p* = 0.092). Given that all the treatment groups were greater than the adjusted *p*-value of 0.0167, we concluded that none of the treatments significantly increased the time to reach a tumor volume of 2000 mm^3^. These results demonstrate that although combination ATR and IMPDH inhibition is efficacious during treatment it does not have a significant survival benefit following the conclusion of treatment. Further investigation into other therapies in combination with ATR and IMPDH inhibition could improve the efficacy and durability of this treatment.

## Discussion

We confirmed the previously identified ST-MYCL-Tip60 target gene, IMPDH2, as a vulnerability in MCC. Using a panel of established and patient-derived cell lines encompassing both MCCP and MCCN subtypes, we show MCC lines were sensitive to IMPDH inhibition and confirmed the rapid depletion of GMP nucleotides as the dominant effect of IMPDH inhibition using LC-MS and guanosine-based rescue experiments. Furthermore, we confirmed by functional and genetic perturbations in multiple cell lines that p53 enhances sensitivity to IMPDH inhibition but was not strictly required for cytotoxicity. We observed that rapid loss of nascent DNA, but not RNA synthesis, upon IMPDH inhibition correlated with the accumulation of RS markers and apoptotic cell death. Specifically, MPA treatment strongly upregulated phosphorylated CHK1 indicating the activation of the DNA damage response (DDR). Similarly, p53 was activated in response to MPA as evidenced by increased levels of p53 phosphorylation and p53 downstream targets p21 and PUMA increased. Furthermore, activation of ATR was the major contributor to the DDR to MPA treatment. From this, we conclude DNA RS induces DNA damage kinase-dependent p53 activation in response to IMPDH inhibition.

Loss of DNA synthesis following IMPDH inhibition is the predominant effect in MCC cell lines, which differs from the impairment of ribosome biogenesis previously reported for other cell lines and confirmed in this study. IMPDH inhibition led to total loss of nascent dNTP incorporation in MCC cell lines, while nascent NTP incorporation and pre-rRNA transcripts were not significantly affected; whereas, in U87MG and NCI-H524 cells, pre-rRNA levels were significantly reduced while DNA synthesis was not completely abolished over the course of 24 h. We find this difference particularly striking between the MCC lines and the small cell lung cancer NCI-H524 cell line given that these cell lines are derived from high grade neuroendocrine cancers.^20^^,^^34^ Future studies will be needed to understand why similar neuroendocrine cancers have different dependencies on GMP nucleotide pools. Further investigation would also be warranted whether neuroendocrine cancers where ribosome biogenesis is impaired would respond to combination treatment of IMPDH and ATR inhibitors which predominantly generates RS leading to cellular cytotoxicity as demonstrated in the present study.

We exploited the IMPDH inhibition-dependent generation of RS in combination with an ATR inhibitor to enhance cytotoxicity. Utilizing chromatin flow cytometry, we observed the rapid exhaustion of chromatin-bound RPA32 followed by a dramatic increase in chromatin-bound γH2AX in dual-treated cells. This induction of RC by co-treatment with an ATR inhibitor increases sensitivity for less responsive MCC cell lines. Although treatment with an IMPDH inhibitor alone induced RS, RPA32 was not exhausted nor was the RC threshold reached. MCC chemotherapy regimens are typically comprised of a topoisomerase II poison such as etoposide combined with an alkylating agent (cisplatin),^11^^,^^12^^,^^14^ which despite being known to inflict RS are unlikely to induce RC as single agents. As such, combining IMPDH or other RS-inducing chemotherapies with ATR inhibition may improve efficacy. Furthermore, the combined efficacy of IMPDH and ATR inhibition warrants further investigation more broadly in cancer.

There is an urgent clinical need to identify novel treatment options for MCC. MCC is a rapidly growing cancer with a high risk for recurrence after initial treatment with rapid development of resistance. Although PD-1/PD-L1 targeted therapies induce responses in approximately half of MCC patients, there are few treatment options for those patients who are initially resistant or develop secondary resistance to ICI.^15^^,^^16^ Here, we demonstrated that MCC is highly susceptible to RS and this susceptibility has the potential to be exploited therapeutically. There is resurging interest in targeting ATR and other DDR proteins in cancer due to DDR inhibitors causing an immunogenic cell death *in vitro* that could potentially enhance ICI therapy.^46^ It is possible that combination of IMPDH and ATR inhibitors would create extensive DNA damage in tumor cells, making them more susceptible to effector functions of immune cells reenergized by immunotherapy. There is also potential for dual IMPDH and ATR inhibition as a treatment in refractory MCC and in combination with other DNA damage-inducing agents like radiotherapy, which is also standard in the treatment of MCC. Treatment plans need to be carefully designed given the activity of IMPDH inhibitors as immunosuppressants.^47^

## Resource availability

### Lead contact

Further information and requests for resources and reagents should be directed to James A. DeCaprio (James_DeCaprio@dfci.harvard.edu).

### Materials availability

Cell lines or plasmids generated in this manuscript are available upon request.

### Data and code availability

All data reported in this paper will be shared by the lead contact upon request. This paper does not report original code. Any additional information required to reanalyze the data reported in this paper is available from the lead contact upon request.

## Acknowledgments

We thank William G. Kaelin, Jr. (Dana-Farber Cancer Institute) for use of LC-MS. We thank all the members of the DeCaprio laboratory for thoughtful discussions and reagent sharing.

This work was supported in part by the US Public Health Service grants R35CA232128 and P01CA203655, the Bridge Project, a partnership between the 10.13039/100016872Koch Institute for Integrative Cancer Research at MIT and the Dana-Farber/Harvard Cancer Center, and by the CaNCURE Award 5R25CA174650.

## Author contributions

T.C.F. and J.L.S. designed and performed experiments in addition to writing the manuscript. A.C.W. assisted in acquiring and analyzing the LC-MS data in addition to writing the corresponding methods. V.A. contributed to acquiring the Nutlin-3a data and in experimental design. S.G. contributed to acquiring qPCR data. K.M.S. and P.C.G. optimized and performed all animal studies in this study. J.A.D. assisted in conceptualization and supervision of the study.

## Declaration of interests

J.A.D. has received research support from Rain Therapeutics, Inc. and Kymera Therapeutics, Inc. He has consulted for Takeda, Inc. and Mariana Oncology, Inc. T.C.F. is currently an employee of AstraZeneca. The remaining authors declare no competing interests.

## STAR★Methods

### Key resources table

**Table:** 

REAGENT or RESOURCE	SOURCE	IDENTIFIER
**Antibodies**		

PARP-1	Cell Signaling Technology	CAT# 9542; RRID: AB_2160739
pp53 (Ser15)	Cell Signaling Technology	CAT# 9284; RRID: AB_331464
p53 (DO-1)	Santa Cruz Biotechnology	CAT# sc-126; RRID: AB_628082
PUMA	Cell Signaling Technology	CAT# 4976; RRID: AB_2064551
p21 Waf1/Cip1 (12D1)	Cell Signaling Technology	CAT# 2947; RRID: AB_823586
Caspase-3	Cell Signaling Technology	CAT# 9662; RRID: AB_331439
β-Actin (C4)	Santa Cruz Biotechnology	CAT# sc-47778; RRID: AB_626632
TBP	Cell Signaling Technology	CAT# 8515; RRID: AB_10949159
Vinculin (hVIN-1)	Sigma Aldrich	CAT# V9131; RRID: AB_477629
pKAP1 (Ser824)	Bethyl Laboratories Inc.	CAT# A300-767A; RRID: AB_669740
KAP1	Bethyl Laboratories Inc.	CAT# A300-274A; RRID: AB_185559
pCHK2 (Thr68; C13C1)	Cell Signaling Technology	CAT# 2197; RRID: AB_2080501
CHK2 (1C12)	Cell Signaling Technology	CAT# 3440; RRID: AB_2229490
pCHK1 (D7H2; Ser317)	Cell Signaling Technology	CAT# 8191; RRID: AB_10859365
CHK1 (2G1D5)	Cell Signaling Technology	CAT# 2360; RRID: AB_2080320
pRPA32 (E5A2F; Ser8)	Cell Signaling Technology	CAT# 54762; RRID: AB_2799471
RPA32 (E8X5P)	Cell Signaling Technology	CAT# 35869; RRID: AB_2799086
γH2AX (clone JBW301; Ser139)	Sigma Aldrich	CAT# 05-636; RRID: AB_309864
CDC45 (D7G6)	Cell Signaling Technology	CAT# 11881; RRID: AB_2715569
PCNA (PC10)	Cell Signaling Technology	CAT# 2586; RRID: AB_2160343
Anti-mouse IgG-PE conjugate	Cell Signaling Technology	CAT# 8887;RRID: AB_2797678
Anti-rabbit IgG-AF647 conjugate	Cell Signaling Technology	CAT# 4414; RRID: AB_10693544
Anti-mouse IgG-HRP	Bethyl Laboratories Inc.	CAT# A90-116P; RRID: AB_67183
Anti-rabbit IgG-HRP	Bethyl Laboratories Inc.	CAT# A120-113P; RRID: AB_10755117

**Chemicals, peptides, and recombinant proteins**		

Mycophenolic acid (MPA)	Sigma Aldrich	CAT# M5255
Mycophenolate mofetil (MMF/CellCept)	Selleck	CAT# S1501
AVN-944 (VX-944)	Cayman Chemical	CAT# 21284
Guanosine	Santa Cruz Biotechnology	CAT# sc-218575
Guanosine monophosphate (as standard)	Sigma Aldrich	CAT# G8377
Inosine monophosphate (as standard)	Sigma Aldrich	CAT# 57510
Adenosine monophosphate (as standard)	Sigma Aldrich	CAT# 01930
Doxycycline (DOX)	Takara Bio	CAT# 631311
5-Ethynyl-2′-deoxyuridine (EdU)	Click Chemistry Tools	CAT# 1149
5-Ethynyl-uridine (5EU)	Click Chemistry Tools	CAT# 1261
Aphidicolin	Santa Cruz Biotechnology	CAT# 201535
Hydroxyurea	Santa Cruz Biotechnology	CAT# 29061
Actinomycin D	Santa Cruz Biotechnology	CAT# 200906
Berzosertib (VE-822)	Selleck	CAT# S7102
KU55933	Selleck	CAT# S1092
AZD7648	Selleck	CAT# S8843
Puromycin	Takara Bio	CAT# 631305
Nutlin-3a	Selleck	CAT# S8059

**Critical commercial assays**		

FITC Annexin V Apoptosis Detection Kit I	BD Biosciences	CAT# 556547
PE Apoptosis Detection Kit I	BD Biosciences	CAT# 559763
RNeasy Mini Kit	Qiagen	CAT# 74104
Qiashredder	Qiagen	CAT# 79656
High-Capacity RNA-to-cDNA Kit	Thermo Scientific	CAT# 4387406
Brilliant III Ultra-Fast SYBR Green QPCR Master Mix	Agilent	CAT# 600882
CellTiter-Glo 2.0	Promega	CAT# G9241

**Experimental models: Cell lines**		

MKL-1	Gift from Masahiro Shuda, University of Pittsburgh, PA	N/A
WaGa	Gift from Jürgen Becker, University Duisburg-Essen, Germany	N/A
PeTa	Gift from Roland Houben, University of Wuerzburg, Germany	N/A
MS-1	Gift from Masahiro Shuda, University of Pittsburgh, PA	N/A
MCCN290	Gift from Catherine Wu, Dana-Farber Cancer Institute, MA)	N/A
MCCN350	Gift from Catherine Wu, Dana-Farber Cancer Institute, MA	N/A
MCCP301	Gift from Catherine Wu, Dana-Farber Cancer Institute, MA)	N/A
MCCP336	Gift from Catherine Wu, Dana-Farber Cancer Institute, MA	N/A
MCCN428	*de novo* isolated	N/A
HEK 293T	ATCC	N/A
U87MG	ATCC	HTB-14
NCI-H524	ATCC	CRL-5831
p53 knockout MKL-1 cell lines	Generated by T.C.F.; Ananthapadmanabhan et al. 2023	N/A
P53DD & eGFP WaGa cell lines	Generated by T.C.F.; Ahmed et al. 2022	N/A

**Recombinant DNA**		

pLIX-402	Addgene	CAT# 91700
pLentiCRISPRv2	Addgene	CAT# 52961
psPAX2	Addgene	CAT# 12260
pMD2.G	Addgene	CAT# 12259
pDONR221_eGFP	Addgene	CAT# 25899
pbabe-hTERT+p53DD	Addgene	CAT# 11128

**Software and algorithms**		

GraphPad Prism v10	GraphPad	N/A
FlowJo v10.8.1	FlowJo	N/A
ElMaven v.0.2.4	Elucidata	N/A

### Experimental model and subject details

MKL-1 and MS-1 cell lines were a gift from Masahiro Shuda (University of Pittsburgh, PA). The WaGa cell line was a gift from Jürgen Becker (University Duisburg-Essen, Germany). The PeTa cell line was a gift from Rolan Houben (University of Würzburg, Germany). The HEK 293T, U87MG, and NCI-H524 cell lines were acquired from the American Type Culture Collection (ATCC). The p53 knockout MKL-1 cell lines^8^ and WaGa cells with inducible eGFP or p53DD^9^ were generated by T.C.F. and are described in the manuscripts referenced in the key resources table. The MCCN290, MCCP301, MCCP336, and MCCN350 PDCLs were a gift from Catherine Wu (Dana-Farber Cancer Insitute, MA). Detailed culturing conditions are detailed in the methods.

### Method details

#### Tissue culture and cell line generation

Established MCC cell lines and NCI-H524 cells were cultured in RPMI-1640 supplemented with 10% FBS, 1% PenStrep (Thermo Scientific), and 1% GlutaMAX (Thermo Scientific). More details on MCC cell lines can be found in Table S1. HEK 293T cells were cultured in DMEM with 10% FBS, 1% PenStrep, and 1% GlutaMAX. U87MG cells were cultured in EMEM with 10% FBS, 1% PenStrep, and 1% GlutaMAX. PDCLs were cultured in Neurocult NS-A medium with 10% NS-A supplement, 20 ng/mL FGF (StemCell Technologies), 20 ng/mL EGF (Thermo Scientific), 0.0002% heparin (StemCell Technologies), and 1% PenStrep. All cells were incubated at 37°C in 5% CO_2_.

#### Viability assays

1000 cells were plated into opaque 96-well tissue culture plates and overlaid with drug to achieve the final concentration listed in a total volume of 100 μL. For the inducible p53DD and eGFP cell lines, cells were pretreated with 1 μg/mL DOX for 24 h at identical cell concentrations and volumes. The next day, 100 μL of 2x drug concentration were overlaid to generate the final drug concentrations. For the guanosine rescue experiments, drug and guanosine were added simultaneously. In each case established cell lines and PDCLs were incubated for 3 or 5 days, respectively. After incubation, CellTiter-Glo 2.0 reagent was added as recommended (Promega) and luminescence was measured on an M200 Infinite plate reader (Tecan). Alternatively, viable cells were counted on a hemocytometer after addition of equal volumes of cells and 0.4% trypan blue stain.

#### LC-MS

WaGa cells were plated at 500k per mL into a 12-well dish and incubated for 24 h before treatment. The next day, drugs were added to the specified final concentrations and returned to the incubator for the listed time. To harvest, cells were washed once in a normal saline solution (0.7 g/L NaCl) and metabolites were extracted via addition of ice-cold (−20°C) 80% MeOH followed by vortexing for 20 min at 4°C and centrifugation at maximum speed for 10 min at 4°C. Metabolite samples were dried overnight using a CentriVap (Labconco) concentrator. LC-MS analysis was performed using a Q Exactive orbitrap mass spectrometer (Thermo Fisher) equipped with an Ion Max source and heated electrospray ionization (HESI) probe, which was coupled to a Vanquish UHPLC system (Thermo Fisher). External mass calibration was performed every 7 days using standard calibration mixture. The dried metabolites were resuspended in a 1:1 mixture of 20 mM ammonium carbonate and 0.1% ammonium hydroxide (Solvent A) and 100% acetonitrile (Solvent B) and were chromatographically separated by injection of 10 μL of resuspended sample into a SeQuant ZIC-pHILIC column (2.1 mm × 150 mm, 5 μm particle size; EMD Millipore) equipped with a guard column (2.1 × 20 mm, 5 μm particle size; EMD Millipore). The gradient was 0–20 min, linear gradient from 80% to 20% B; 20–20.5 min, linear gradient from 20% to 80% B; 20.5 to 28 min, 80% B. The flow rate was 150 μL/min. MS full scan was performed in negative ionization mode with m/z range of 70–1000, resolution of 70,000, AGC target 1e6, and maximum integration time of 20 msec. The spray voltage was held at 3.0 kV, heated capillary at 275°C, and HESI probe at 350°C. The sheath gas flow rate was 40 units, the auxiliary gas flow was 15 units, and the sweep gas flow was 1 unit. All LC-MS data were analyzed using ElMaven (v.0.2.4, Elucidata). Compound identification was based on exact mass and retention time matching to commercial standards. Metabolite data were normalized to control conditions.

#### AV and PI or DAPI cytotoxicity assay

Cells were plated at 500k per mL into 12-well dishes and incubated for 24 h before treatment. For the inducible p53DD or eGFP cell lines, cells were pretreated with 1 μg/mL DOX for 24 h in identical conditions. The next day, drug and/or guanosine were added to the specified final concentrations and returned to the incubator for 48 h. For the AV/PI stain, the FITC Annexin V Apoptosis Detection Kit I (BD Biosciences) was used as per protocol. For the AV/DAPI stain, the PE Apoptosis Detection Kit I (BD Biosciences) was used as recommended except that PI was substituted for 1 μg/mL DAPI. Cells were filtered through a 70 μm filter before processing on an LSR Fortessa (BD Biosciences) and analysis via FlowJo.

#### Immunoblots

Cells were plated at 500k cells per mL into 10 cm dishes and incubated for 24 h before treatment. The inducible p53DD or eGFP WaGa cell lines were pretreated with 1 μg/mL DOX for 24 h in identical conditions. The next day, drug and guanosine were added to the listed final concentrations and returned to the incubator for 24 or 72 h for the WaGa and MKL-1 cell lines, respectively. After treatment, cells were washed once with PBS and lysed in RIPA buffer supplemented with protease and phosphatase inhibitors (each 1:100, EMD Millipore) in addition to 2-betamercaptoethanol (1:10,000) for 15 min on ice. The resulting lysate was cleared and quantitated via Bradford assay (BioRad). Equal amounts of protein were loaded onto 4–20% polyacrylamide gels and run in a Criterion Cell (BioRad). Protein was transferred onto a nitrocellulose membrane (BioRad) and blocked in 5% non-fat milk in TBST for 1 h at room temperature. Primary antibodies were used at the following concentrations overnight in 5% milk in TBST at 4°C with agitation: PARP-1 (1:1000), pKAP1 (1:1000), KAP1 (1:1000), pCHK1 (1:1000), CHK1 (1:1000), pCHK2 (1:1000), CHK2 (1:1000), pp53 (1:1000), p53 (1:1000), pRPA32 (1:1000), RPA32 (1:10 000), PUMA (1:1000), γH2AX (1:1000). eGFP (1:1000), p53DD (1:500), CASP3 (1:500), β-actin (1:5000), TBP (1:1000), and vinculin (1:30 000). After washing with TBST, anti-mouse or anti-rabbit secondary antibodies conjugated to HRP were incubated at 1:1000 for 1 h at room temperature in 5% milk in TBST with agitation. After the final incubation, membranes were washed and incubated with either Immobilon (EMD Millipore) or Clarity (BioRad) chemiluminescent substrates and luminescence detected on the G-box system (Syngene). Image analysis was performed in ImageJ. For blots that were reblotted, blots were washed with TBST once and then stripped by incubation with Restore PLUS western blot stripping buffer (Thermo Scientific) for 10 min. Blots were then washed three times with TBST and blocked with 5% milk in TBST for 1 h at room temperature. Steps for primary, secondary, and analysis by chemiluminescence were performed following the protocol described above.

#### RTqPCR method for RNA polymerase activity

Cells were plated at 500k cells per mL into 6-well dishes and incubated for 24 h before treatment. The next day, MPA (1 μM) and guanosine (10 μM) were added and the plates were returned to the incubator for 24 or 72 h for WaGa and MKL-1 cells, respectively. After treatment, cells were washed once with PBS and RNA was extracted via TRIzol (Thermo Scientific) as recommended before quantitation with a Nanodrop (Thermo Scientific). 2 μg of extracted RNA was reversed transcribed via the High-Capacity RNA-to-cDNA Kit (Thermo Scientific) and the resulting cDNA was diluted 1:10 in RNAase-free water. RT-qPCR was performed on the diluted cDNA using the primers listed in the Table S2 with the Brilliant III Ultra-Fast SYBR Green QPCR Master Mix (Agilent) on the MxAria (Agilent) system. Relative expression was calculated utilizing the ΔΔCt method with the geometric mean of β-actin and β-2-microglobulin as controls.

NCI-H524, WaGa, & MKL-1 Cells were plated at 500k cells per mL into 6-well dishes and incubated for 24 h before treatment. U87MG were seeded at 500K cells per 15 cm dish and incubated 24 h before treatment. The next day, MPA (1 μM) and guanosine (10 μM) were added and the plates were returned to the incubator for 4 and 24 h for WaGa cells and 8 and 24 h for MKL-1, U87MG, and NCI-H524 cells. After treatment, cells were washed once with PBS and RNA was extracted using RNeasy mini kit following the manufacturer’s recommendations before quantitation with a Nanodrop (Thermo Scientific). 1 μg of extracted RNA was reversed transcribed via the High-Capacity RNA-to-cDNA Kit (Thermo Scientific). cDNA template was optimized for each primer pair and diluted with RNase Free water to within the linear range of the template dilution curve for RT-qPCR assays. RT-qPCR was performed on the diluted cDNA using the primers listed in the Table S2 with the Brilliant III Ultra-Fast SYBR Green QPCR Master Mix (Agilent) on the MxAria (Agilent) system. Relative expression was calculated utilizing the ΔΔCt method with the geometric mean of β-actin and β-2-microglobulin as controls.

#### Nucleotide labeling flow cytometry

Suspension cells (MKL-1, WaGa, NCI-H524s) were plated at 1 million per mL into 12-well dishes and incubated for 24 h before treatment. Adherent cells (U87MG) were plated at 200k cells/ml in 10 cm plates incubated for 24 h before treatment. The next day, drug and guanosine were added to the specified final concentrations and incubated for the time listed. An hour before harvesting, a final concentration of 10 μM EdU or 1 mM 5EU was added before returning to the incubator. After treatment, cells were washed once with PBS and then fixed in 4% MeOH-free formaldehyde in PBS for 15 min at room temperature. The fixed cells were washed twice with PBS and permeabilized with ice-cold (−20°C) 70% EtOH overnight. The next day, the permeabilized pellets were washed twice with PBS and incorporated nucleotides were labeled via click-chemistry (0.1 mM THPTA, 100 mM sodium ascorbate, 1 mM CuSO_4_, and 1.5 μM AF647 (Click Chemistry Tools)) rotating for 30 min at room temperature. After labeling, cells were washed twice with PBS+1% BSA and then stained with 1 μg/mL DAPI in the dark for 40 min at room temperature. Cells were washed once more, resuspended in PBS+1% BSA, and filtered through a 70 μm filter before processing on an LSR Fortessa (BD Biosciences). The resulting data was analyzed via FlowJo.

#### Chromatin flow cytometry

Cells were plated at 1 million per mL into 12-well dishes and incubated for 24 h before treatment. The next day, drugs were added to the specified final concentrations and incubated for 24 h. After treatment, cells were washed once with PBS before extraction of non-chromatin associated proteins with a freshly prepared, cold (4°C) modified CSK buffer (100 mM sucrose, 100 mL NaCl, 3 mM MgCl_2_, 25 mM HEPES (pH 7.3), 0.5% Triton X-100, protease, and phosphatase inhibitors (each 1:100 v/v, EMD Millipore)) for 5 min on ice. After extraction, MeOH-free formaldehyde (Thermo Scientific) was added to a final concentration of 4% and incubation was continued on ice for 15 min. Permeabilized and fixed cells were washed twice with PBS+1% BSA and incubated overnight in the dark with primary antibodies at the following dilutions: RPA32 (1:250), γH2AX (1:500), CDC45 (1:50), and/or PCNA (1:250). The next day, cells were washed twice with PBS+1% BSA before incubation at room temperature in the dark for 1 h with either anti-mouse PE or anti-rabbit AF647 conjugate secondary antibodies (each 1:1000). After incubation, a final concentration of 2.5 μg/mL DAPI was added for 40 min in the dark at room temperature. Cells were washed one additional time before resuspension in PBS+1% BSA, filtering through a 70 μm filter, and analysis on an LSR Fortessa (BD Biosciences). The resulting data was analyzed via FlowJo.

#### Xenograft models

All xenograft experiments were carried out at the Dana-Farber Cancer Institute Experimental Therapeutics Core. The Dana-Farber Cancer Institute Institutional Animal Care and Use Committee approved all animal studies performed. All animal studies were complaint with The National Institute of Health Guide for the Care and Use of Laboratory Animals. Thirty-four six-to eight-week-old female NOD *scid* gamma mice (Jackson Labs, Bar Harbor, ME) were injected subcutaneously in the right flank with 5 x 10^6^ MKL-1 cells in a 1:1 PBS:Matrigel solution. Biweekly measurements of tumor volumes were taken until tumor volumes reached 117.0–159.5 mm^3^ (mean: 134.9 mm^3^). Animals were then randomly grouped into the following treatment conditions: vehicle control (*N* = 7), MMF (*N* = 8), elimusertib (*N* = 8), MMF & elimusertib (*N* = 11). The treatment period was for 28 days and all treatments were administered through oral gavage. The vehicle groups received 0.9% sodium chloride, 0.5% sodium carboxymethylcellulose, 0.4% benzyl alcohol, in water; pH 3.5 every day during the treatment period. The MMF group received 300 mg/kg MMF every day over the treatment period. The elimusertib group received 30 mg/kg elimusertib twice a day for three days followed by four days off over the treatment period. The combination MMF and elimersertib group received 300 mg/kg MMF every day and 30 mg/kg twice a day for three days followed by four days off over the treatment period. Tumor volumes and body weights were recorded biweekly during the treatment period. Following the conclusion of the treatment period, tumors were allowed to progress until a tumor volume exceeded 2000 mm^3^ was reached, at which animals were euthanized.

### Quantification and statistical analysis

All statistical analyses and IC_50_ calculations were performed in GraphPad Prism v10 with recommended settings. All flow cytometry data was analyzed in FlowJo v10.8.1. All LC-MS data was analyzed in ElMaven v.0.2.4. Details for specific statistical tests are available in the figure legends. In this manuscript, N = number of biological replicates performed for a given assay.

## Supplemental information

Document S1. Figures S1–S6 and Table S1 and S2

Document S2. Appendix containing western blot replicates where available for manuscript figures
