# Supplementary material for: IMPDH inhibition induces DNA replication stress and ATR sensitivity in Merkel cell carcinoma
Source: iScience. 2025 May 2;28(6):112567. doi: 10.1016/j.isci.2025.112567 (PMC12148403; doi:10.1016/j.isci.2025.112567)
Supplement: Document S1. Figures S1–S6 and Table S1 and S2 [file mmc1.pdf]

iScience, Volume *28*

Supplemental information

IMPDH inhibition induces DNA replication stress and ATR sensitivity in Merkel cell carcinoma

Julia L. Schnabel, Thomas C. Frost, Adam C. Wang, Varsha Ananthapadmanabhan, Satvik Gurram, Kara M. Soroko, Prafulla C. Gokhale, and James A. DeCaprio

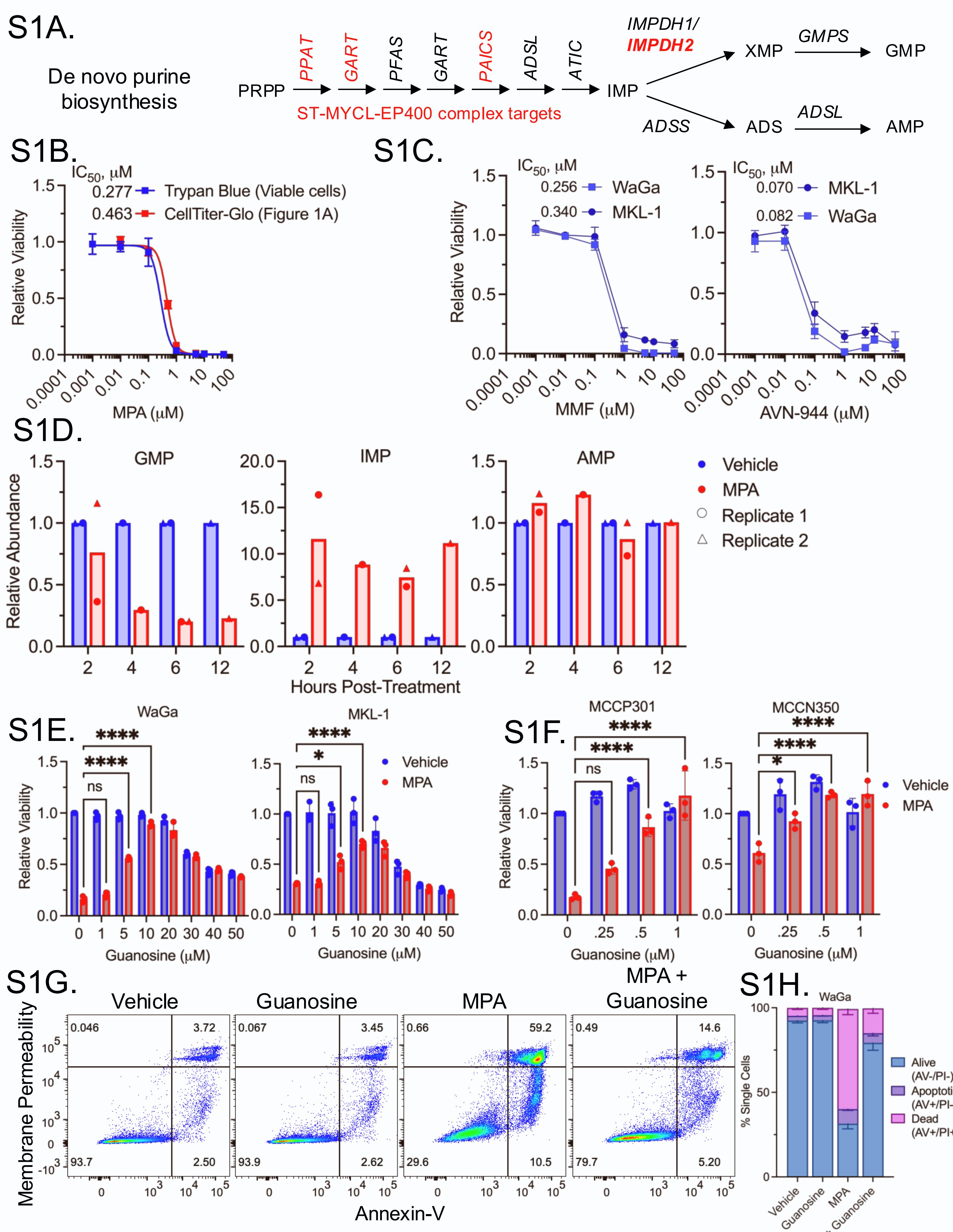

S1A.
De novo purine biosynthesis
PRPP
PPAT
GART
PFAS
GART
PAICS
ADSL
ATIC
IMP
IMPDH1/
IMPDH2
XMP
GMPS
GMP
ADSS
ADS
ADSL
AMP
ST-MYCL-EP400 complex targets
S1B.
IC50, μM
0.277 Trypan Blue (Viable cells)
0.463 CellTiter-Glo (Figure 1A)
Relative Viability
MPA (μM)
S1C.
IC50, μM
0.256 WaGa
0.340 MKL-1
MMF (μM)
IC50, μM
0.070 MKL-1
0.082 WaGa
AVN-944 (μM)
S1D.
GMP
IMP
AMP
Relative Abundance
Hours Post-Treatment
Vehicle
MPA
Replicate 1
Replicate 2
S1E.
WaGa
MKL-1
Relative Viability
Guanosine (μM)
Vehicle
MPA
S1F.
MCCP301
MCCN350
Relative Viability
Guanosine (μM)
Vehicle
MPA
S1G.
Vehicle
Guanosine
MPA
MPA + Guanosine
Membrane Permeability
Annexin-V
S1H.
WaGa
% Single Cells
Alive (AV-/PI-)
Apoptoti (AV+/PI-
Dead (AV+/PI+
Vehicle
Guanosine
MPA
MPA + Guanosine

Figure S1

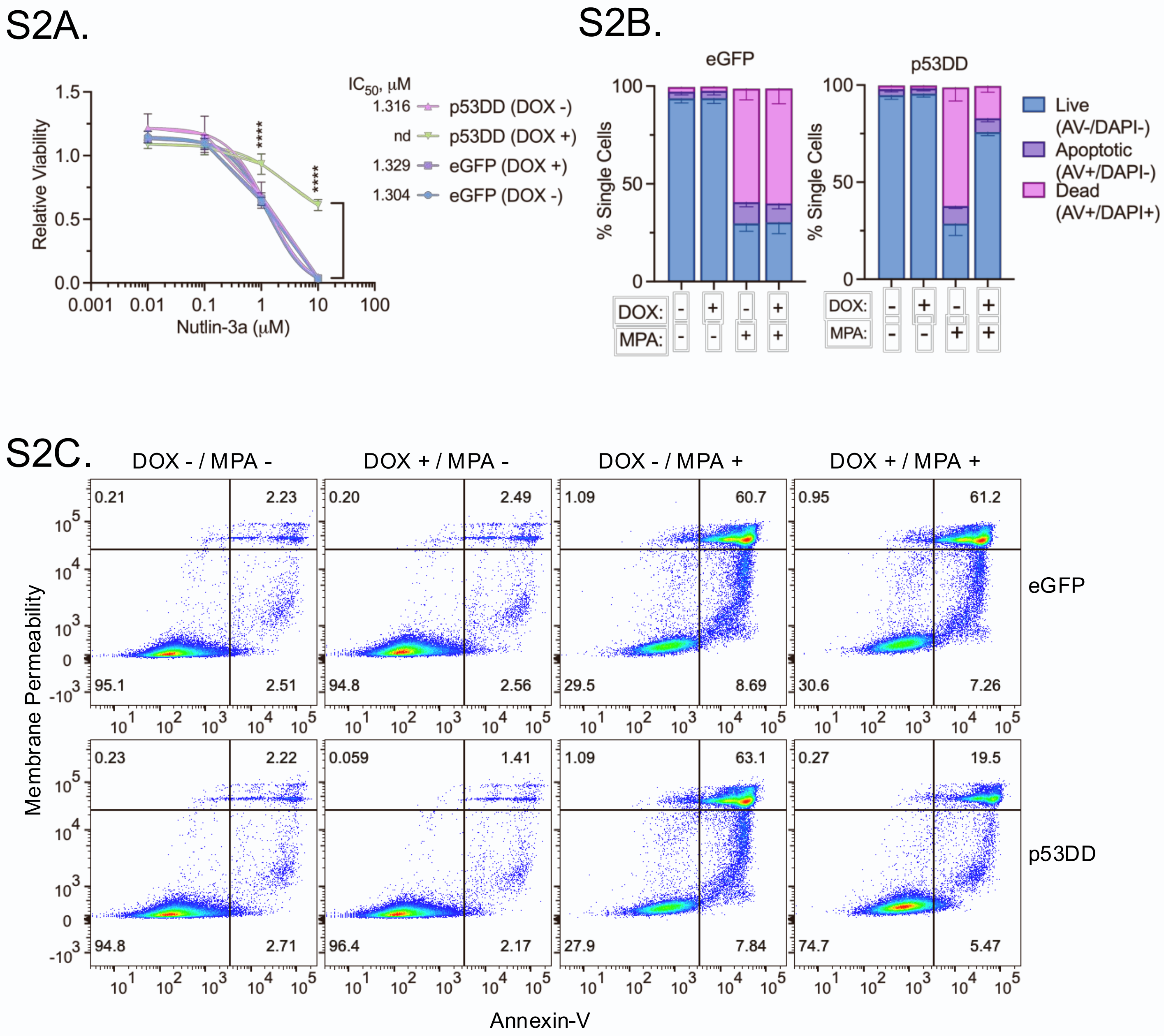

S2A.
IC50, μM
1.316 p53DD (DOX -)
nd p53DD (DOX +)
1.329 eGFP (DOX +)
1.304 eGFP (DOX -)
Relative Viability
Nutlin-3a (μM)
****
S2B.
eGFP
p53DD
% Single Cells
DOX:
MPA:
Live (AV-/DAPI-)
Apoptotic (AV+/DAPI-)
Dead (AV+/DAPI+)
S2C.
DOX - / MPA -
DOX + / MPA -
DOX - / MPA +
DOX + / MPA +
eGFP
p53DD
Membrane Permeability
Annexin-V
0.21 2.23 95.1 2.51
0.20 2.49 94.8 2.56
1.09 60.7 29.5 8.69
0.95 61.2 30.6 7.26
0.23 2.22 94.8 2.71
0.059 1.41 96.4 2.17
1.09 63.1 27.9 7.84
0.27 19.5 74.7 5.47

Figure S2

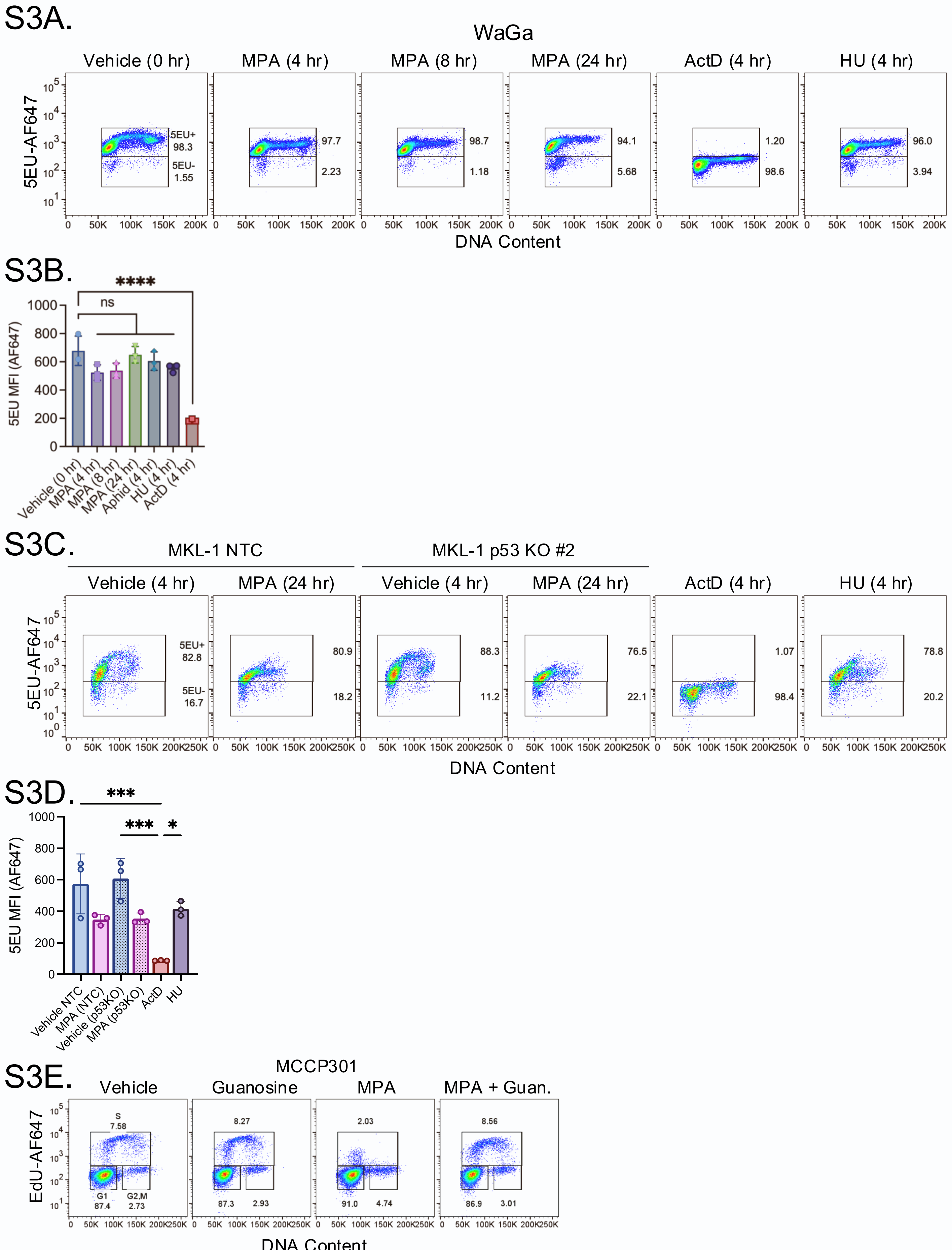
S3A.
WaGa
Vehicle (0 hr)
MPA (4 hr)
MPA (8 hr)
MPA (24 hr)
ActD (4 hr)
HU (4 hr)
5EU-AF647
5EU+ 98.3
5EU- 1.55
97.7
2.23
98.7
1.18
94.1
5.68
1.20
98.6
96.0
3.94
DNA Content
S3B.
****
ns
5EU MFI (AF647)
Vehicle (0 hr)
MPA (4 hr)
MPA (8 hr)
MPA (24 hr)
Aphid (4 hr)
HU (4 hr)
ActD (4 hr)
S3C.
MKL-1 NTC
MKL-1 p53 KO #2
Vehicle (4 hr)
MPA (24 hr)
Vehicle (4 hr)
MPA (24 hr)
ActD (4 hr)
HU (4 hr)
5EU-AF647
5EU+ 82.8
5EU- 16.7
80.9
18.2
88.3
11.2
76.5
22.1
1.07
98.4
78.8
20.2
DNA Content
S3D.
***
***
*
5EU MFI (AF647)
Vehicle NTC
MPA (NTC)
Vehicle (p53KO)
MPA (p53KO)
ActD
HU
S3E.
MCCP301
Vehicle
Guanosine
MPA
MPA + Guan.
EdU-AF647
S 7.58
G1 87.4
G2,M 2.73
8.27
87.3
2.93
2.03
91.0
4.74
8.56
86.9
3.01
DNA Content

Figure S3

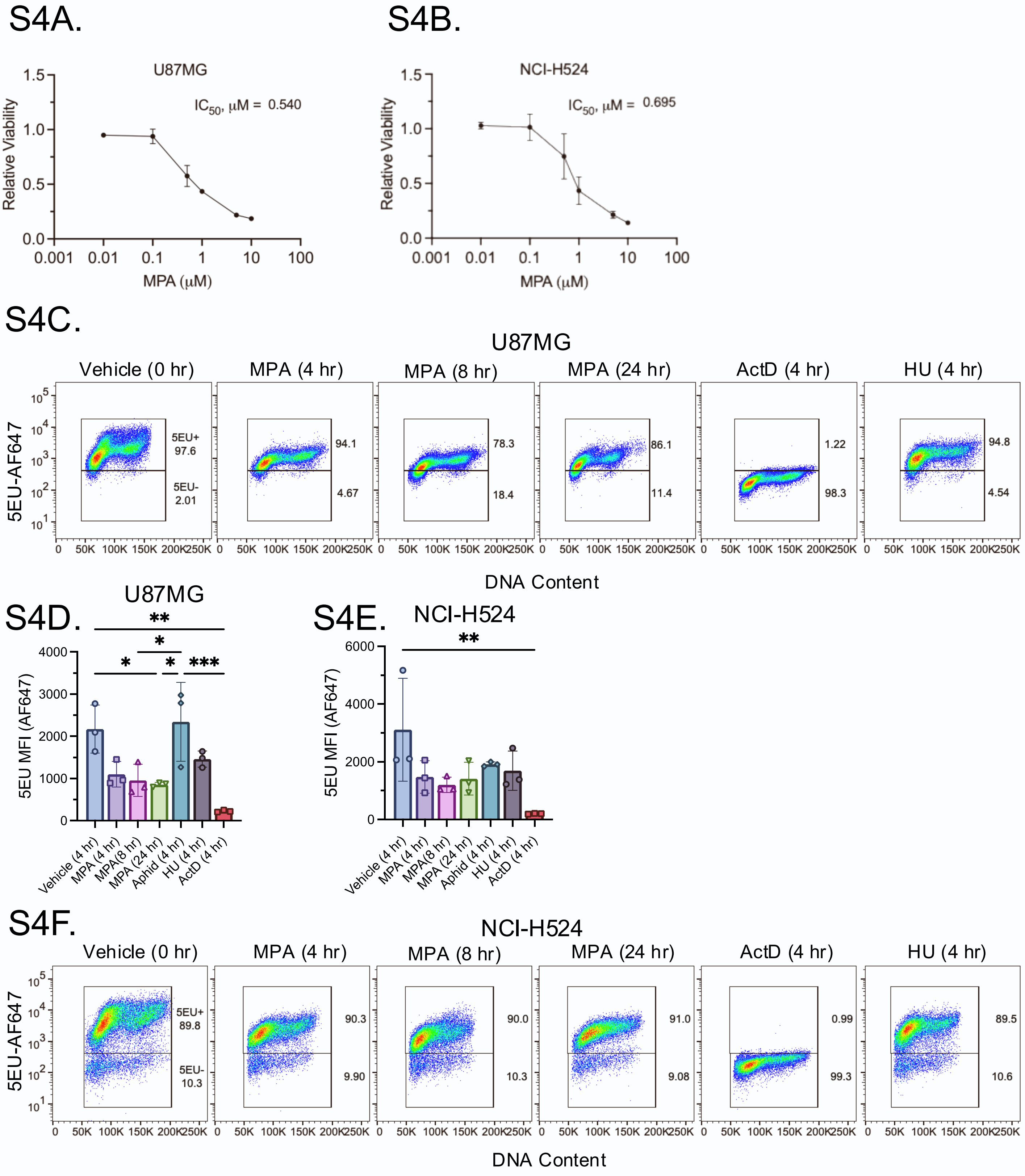

S4A.
U87MG
IC50, μM = 0.540
Relative Viability
MPA (μM)
S4B.
NCI-H524
IC50, μM = 0.695
Relative Viability
MPA (μM)
S4C.
U87MG
Vehicle (0 hr)
MPA (4 hr)
MPA (8 hr)
MPA (24 hr)
ActD (4 hr)
HU (4 hr)
5EU-AF647
5EU+
97.6
5EU-
2.01
94.1
4.67
78.3
18.4
86.1
11.4
1.22
98.3
94.8
4.54
DNA Content
S4D.
U87MG
5EU MFI (AF647)
Vehicle (4 hr)
MPA (4 hr)
MPA(8 hr)
MPA (24 hr)
Aphid (4 hr)
HU (4 hr)
ActD (4 hr)
S4E.
NCI-H524
5EU MFI (AF647)
Vehicle (4 hr)
MPA (4 hr)
MPA(8 hr)
MPA (24 hr)
Aphid (4 hr)
HU (4 hr)
ActD (4 hr)
S4F.
NCI-H524
Vehicle (0 hr)
MPA (4 hr)
MPA (8 hr)
MPA (24 hr)
ActD (4 hr)
HU (4 hr)
5EU-AF647
5EU+
89.8
5EU-
10.3
90.3
9.90
90.0
10.3
91.0
9.08
0.99
99.3
89.5
10.6
DNA Content

Figure S4

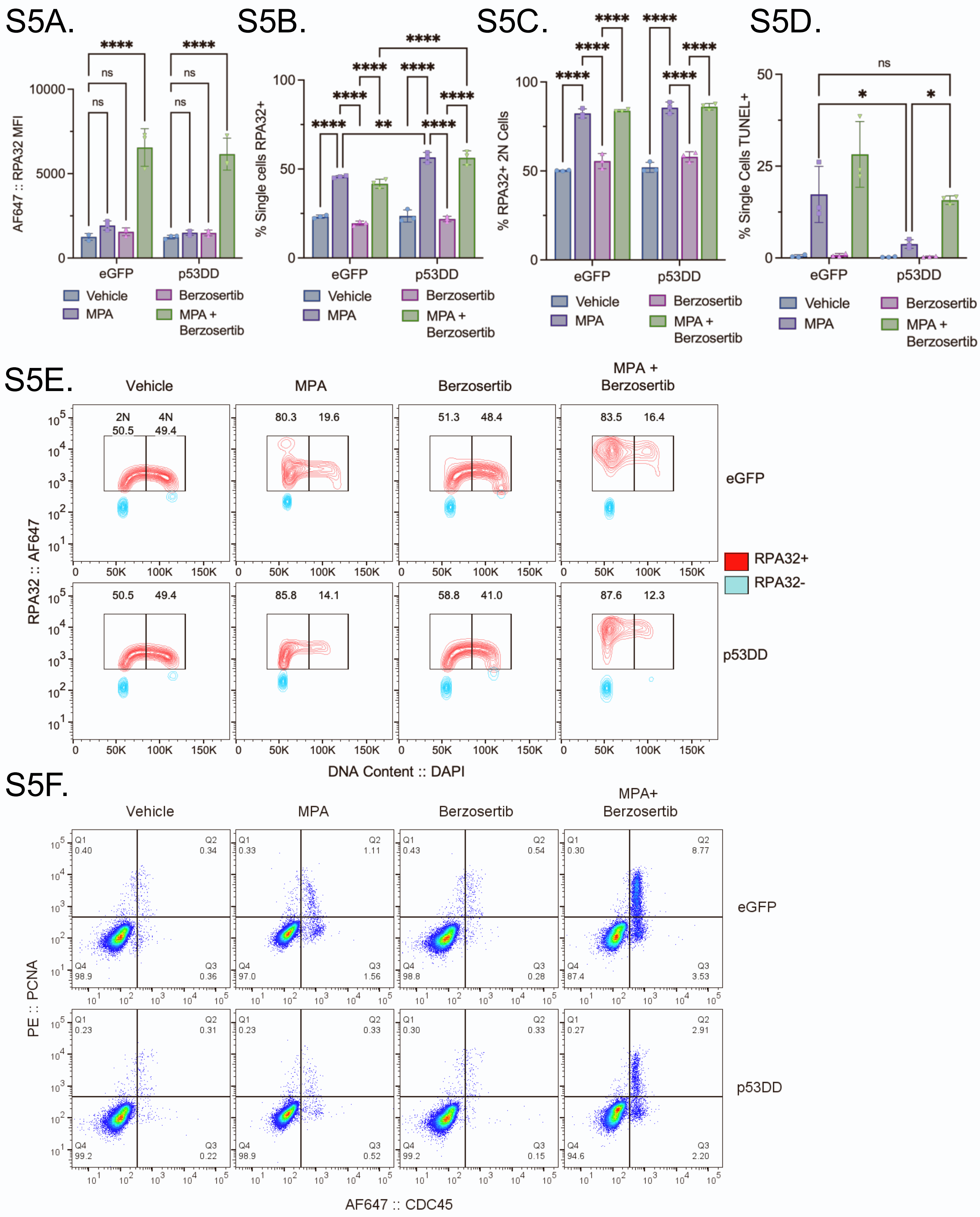
S5A.
AF647 :: RPA32 MFI
eGFP
p53DD
S5B.
% Single cells RPA32+
S5C.
% RPA32+ 2N Cells
S5D.
% Single Cells TUNEL+
Vehicle
MPA
Berzosertib
MPA + Berzosertib
S5E.
Vehicle
MPA
Berzosertib
MPA + Berzosertib
2N
4N
50.5
49.4
80.3
19.6
51.3
48.4
83.5
16.4
50.5
49.4
85.8
14.1
58.8
41.0
87.6
12.3
RPA32 :: AF647
DNA Content :: DAPI
RPA32+
RPA32-
S5F.
Vehicle
MPA
Berzosertib
MPA+ Berzosertib
Q1 0.40
Q2 0.34
Q4 98.9
Q3 0.36
Q1 0.33
Q2 1.11
Q4 97.0
Q3 1.56
Q1 0.43
Q2 0.54
Q4 98.8
Q3 0.28
Q1 0.30
Q2 8.77
Q4 87.4
Q3 3.53
Q1 0.23
Q2 0.31
Q4 99.2
Q3 0.22
Q1 0.23
Q2 0.33
Q4 98.9
Q3 0.52
Q1 0.30
Q2 0.33
Q4 99.2
Q3 0.15
Q1 0.27
Q2 2.91
Q4 94.6
Q3 2.20
PE :: PCNA
AF647 :: CDC45

Figure S5

S6A.

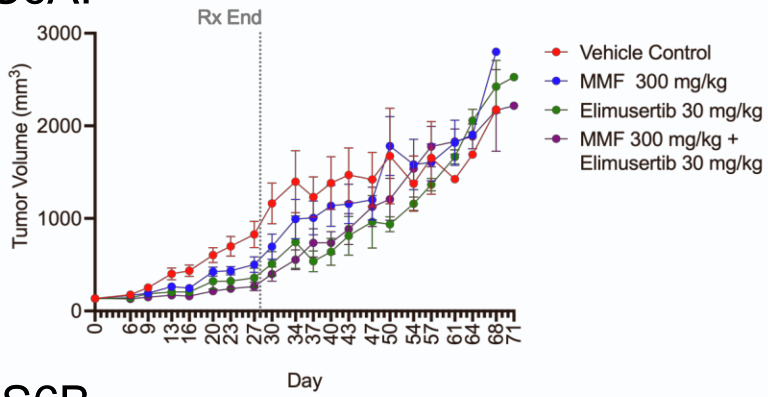

S6B.

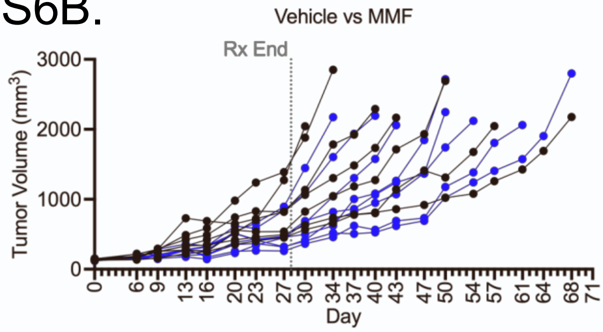

S6C.

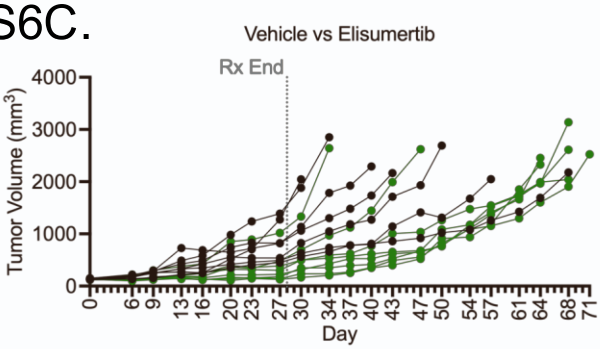

S6D.

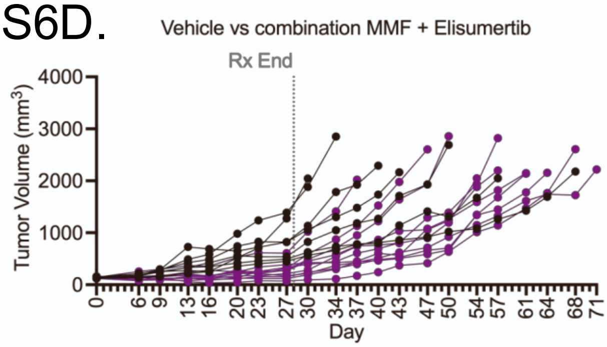

Figure S6

Supplementary Table 1. MCC cell line characteristics

Cell line	Type	Viral Status	p53 Status	Gender	Age at sampling
MKL-1	Established	positive	wildtype	M	26Y
WaGa	Established	positive	wildtype	M	67Y
PeTa	Established	positive	wildtype	M	65Y
MS-1	Established	positive	mutant	F	59Y
MCCP301	PDCL	positive	wildtype	M	67Y
MCCP336	PDCL	positive	wildtype	F	72Y
MCCN290	PDCL	negative	mutant	F	90Y
MCCN350	PDCL	negative	mutant	M	90Y

Supplementary Table 2

RT-qPCR Primers			
**Target**	**Forward Sequence (5'-3')**	**Reverse Sequence (5'-3')**	**Source**
p21 (*CDKN1A*)	GCGACTGTGATGCGCTAAT	GTGGTGTCTCGGTGACAAAG	This manuscript
PUMA (*BBC3*)	GGAGGGTCCTGTACAATCT	CTAATTGGGCTCCATCTCG	This manuscript
β-actin (*ACTB*)	GGACTTCGAGCAAGAGATGG	AGCACTGTGTTGGCGTACAG	Schade et al. 2019
β-2-microglobulin (*B2M*)	TGCTGTCTCCATGTTTGATGTATCT	TCTCTGCTCCCCACCTCTAAGT	Schade et al. 2019
pre-rRNA	GCTCTACCTTACCTACCTGG	TGAGCCATTCGCAGTTTCAC	Huang et al. 2018
ATF4	ATGACCGAAATGAGCTTCCTG	GCTGGAGAACCCATGAGGT	MGH Primer Bank id. 33469973c1
5S rRNA	GGCCATACCACCCTGAACGC	CAGCACCCGGTATTCCCAGG	Huang et al. 2018
