# Supplementary material for: IMPDH inhibition induces DNA replication stress and ATR sensitivity in Merkel cell carcinoma
Source: iScience. 2025 May 2;28(6):112567. doi: 10.1016/j.isci.2025.112567 (PMC12148403; doi:10.1016/j.isci.2025.112567)

ISCIENCE-D-24-12109R1

"Inhibition of IMPDH induces DNA replication stress and sensitivity to ATR inhibition in Merkel cell carcinoma"

Appendix

Contents:

Page 1-2: Uncropped images for p-CHK2 and CHK2 from Figure 5C.
Page 3-4: Western blot replicates for Figure 1 panel E and panel F
Page 5-6: Western blot replicates for Figure 2 panel A and panel B
Page 7-9: Western blot replicates for Figure 5 panel A through panel C
Page 10-11: Western blot replicates for Figure 6 panel A and panel B

Image of p-CHK2 from Figure 5C.

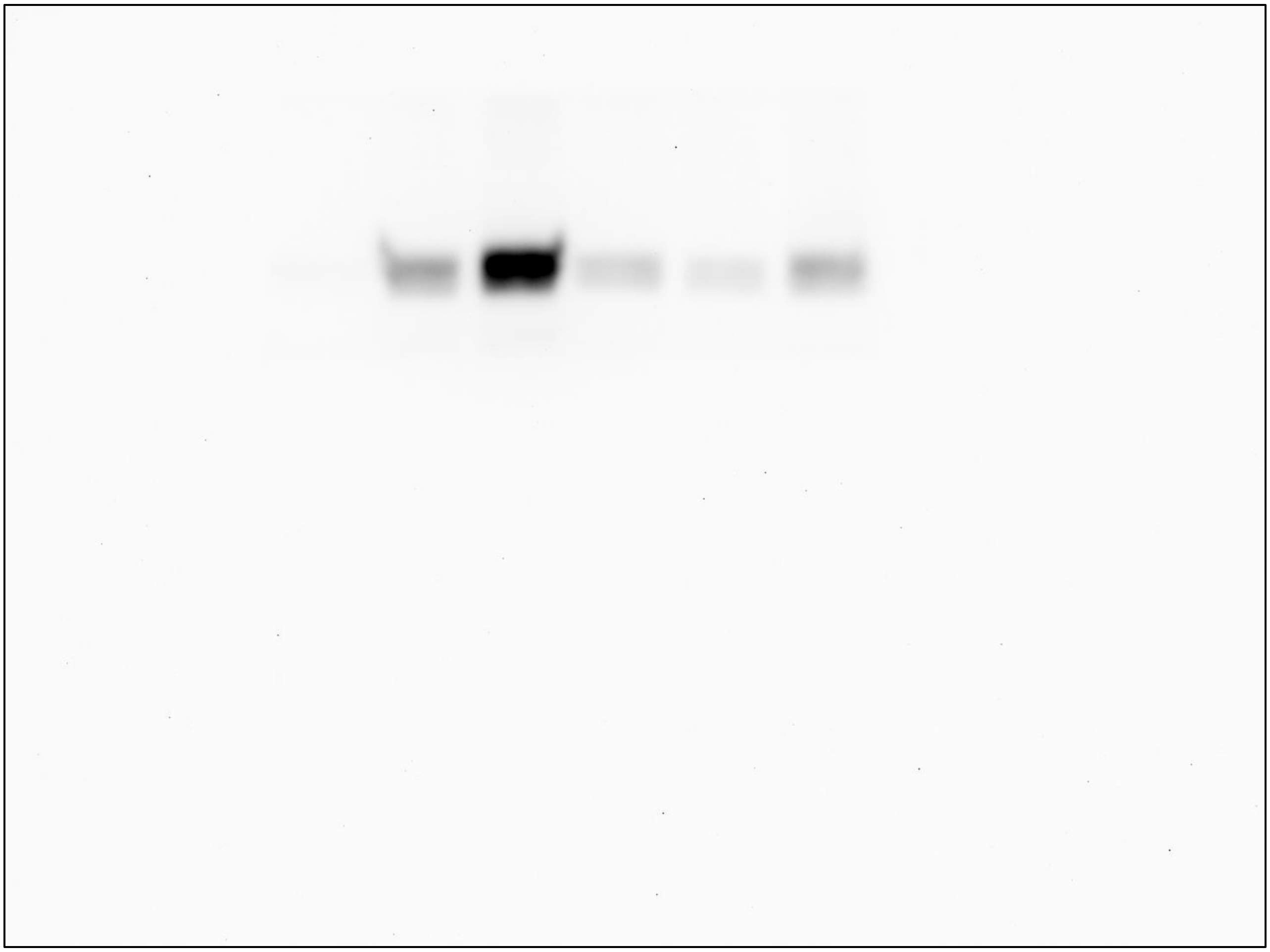

Image of CHK2 from Figure 5C.

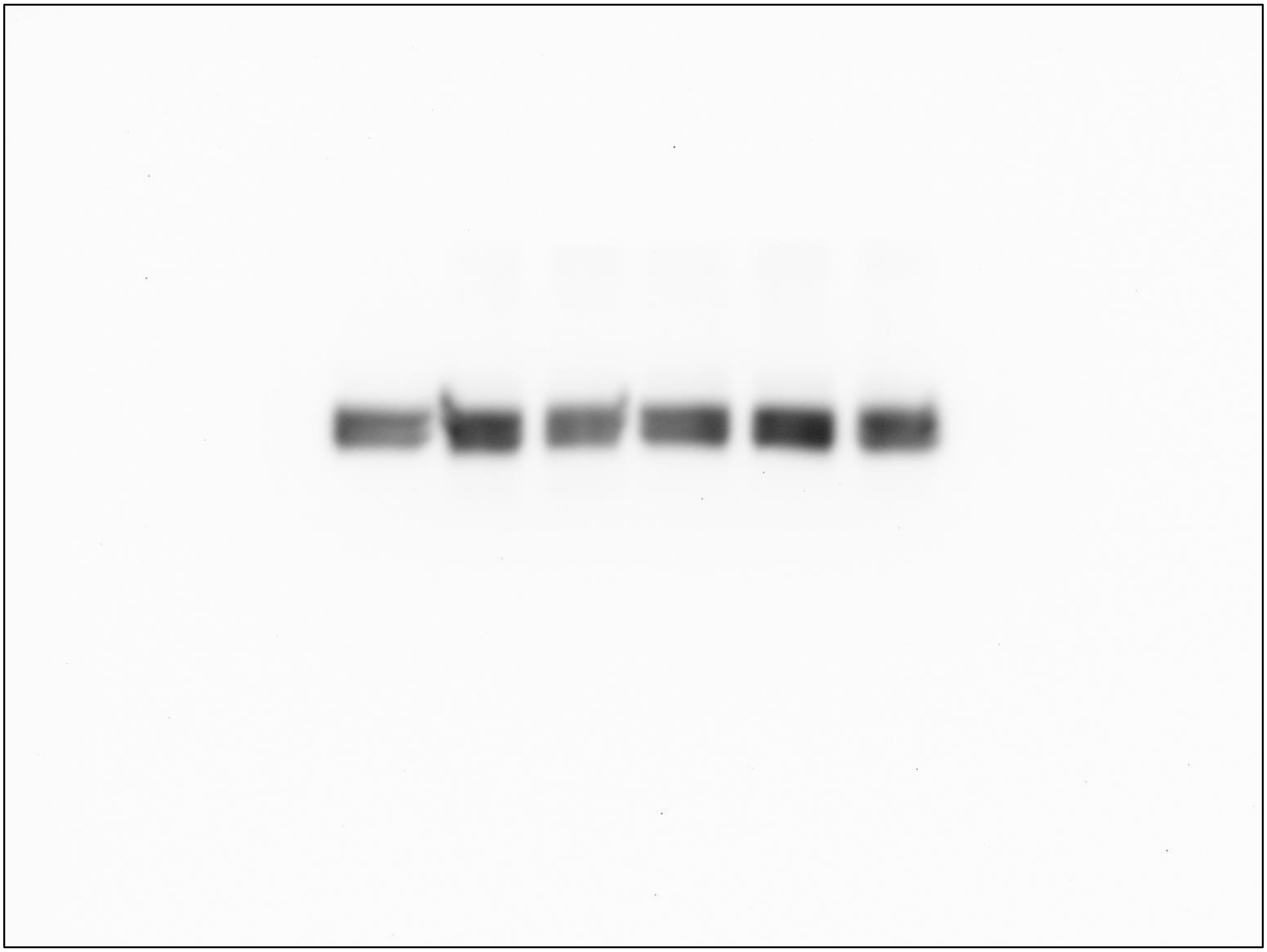

Figure 1E Replicates

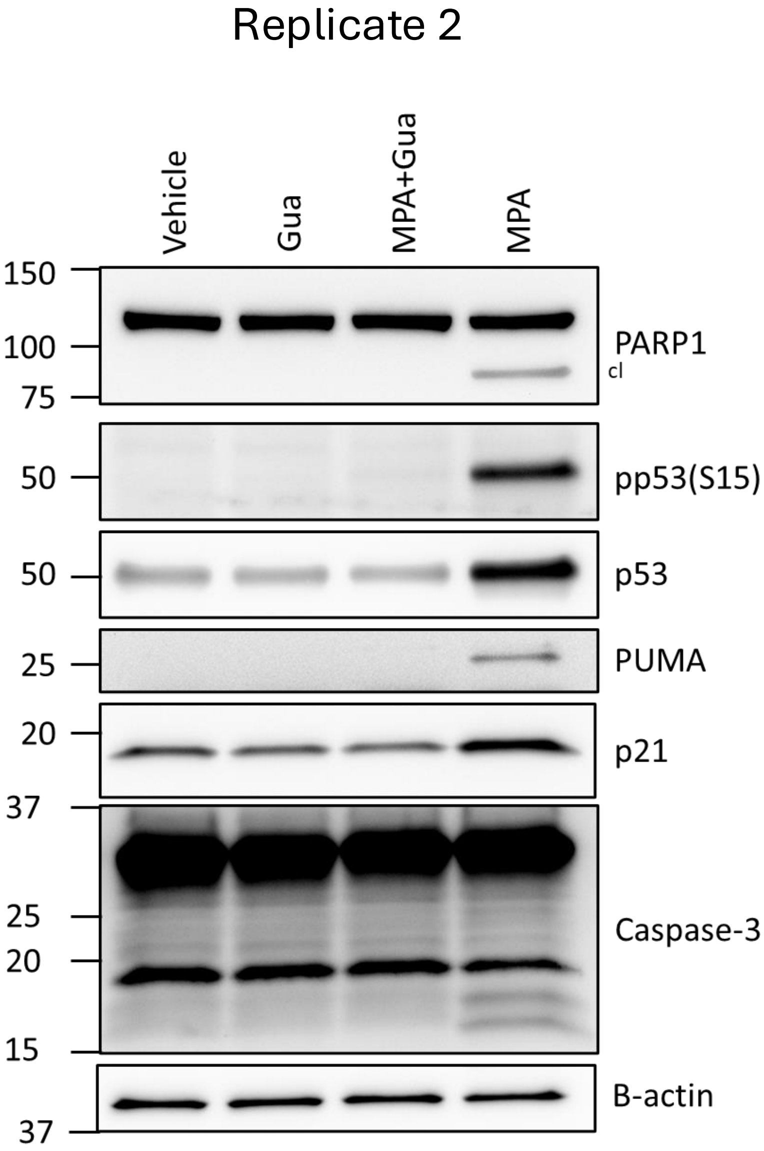

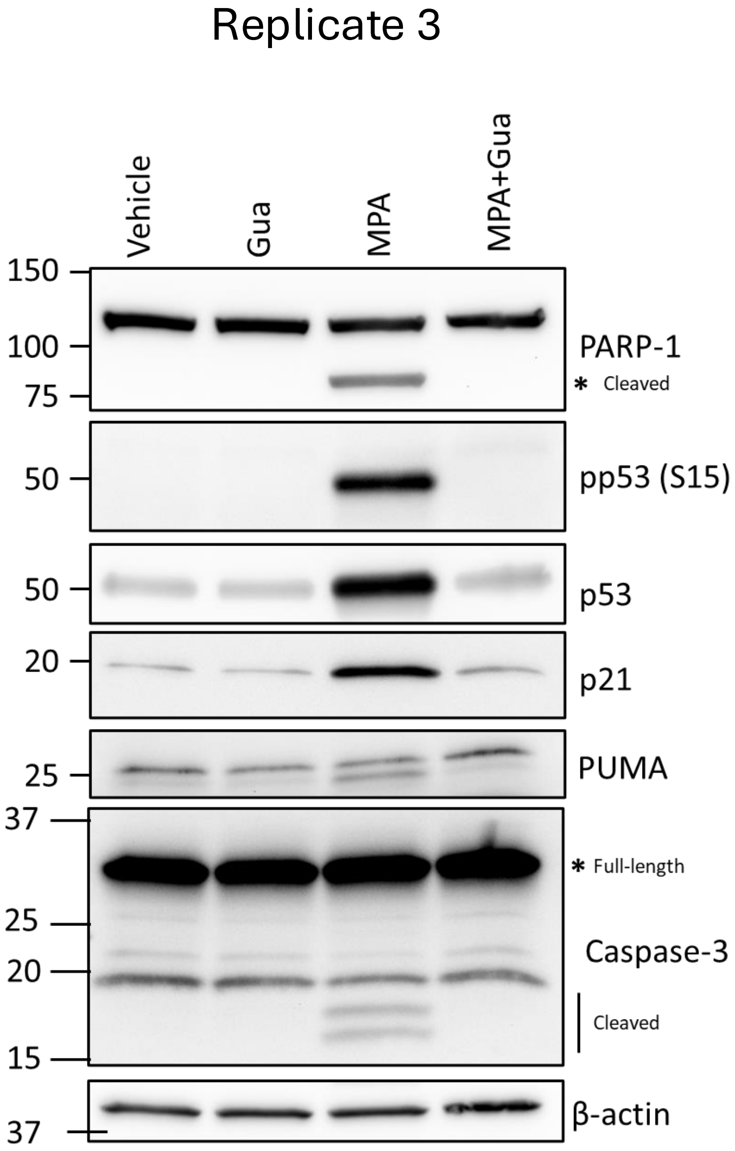

Figure 1F Replicates

Replicate 2

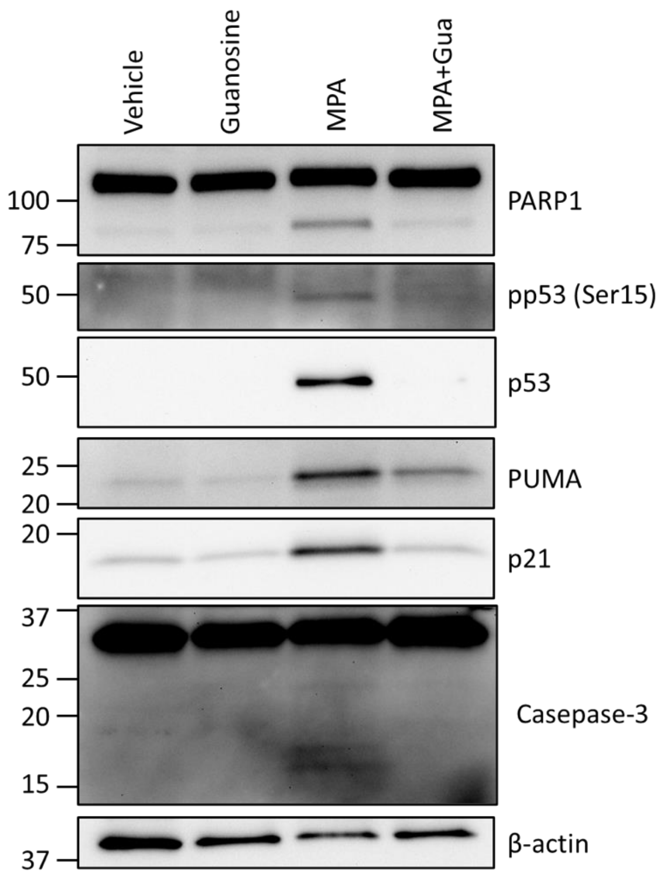

Replicate 3

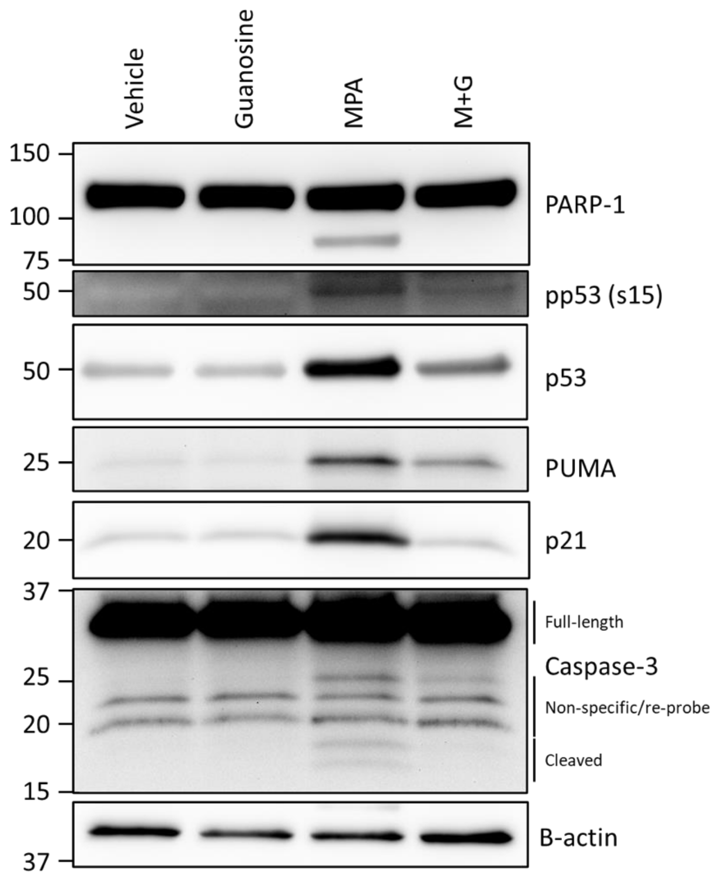

Figure 2A Replicates

Replicate 2

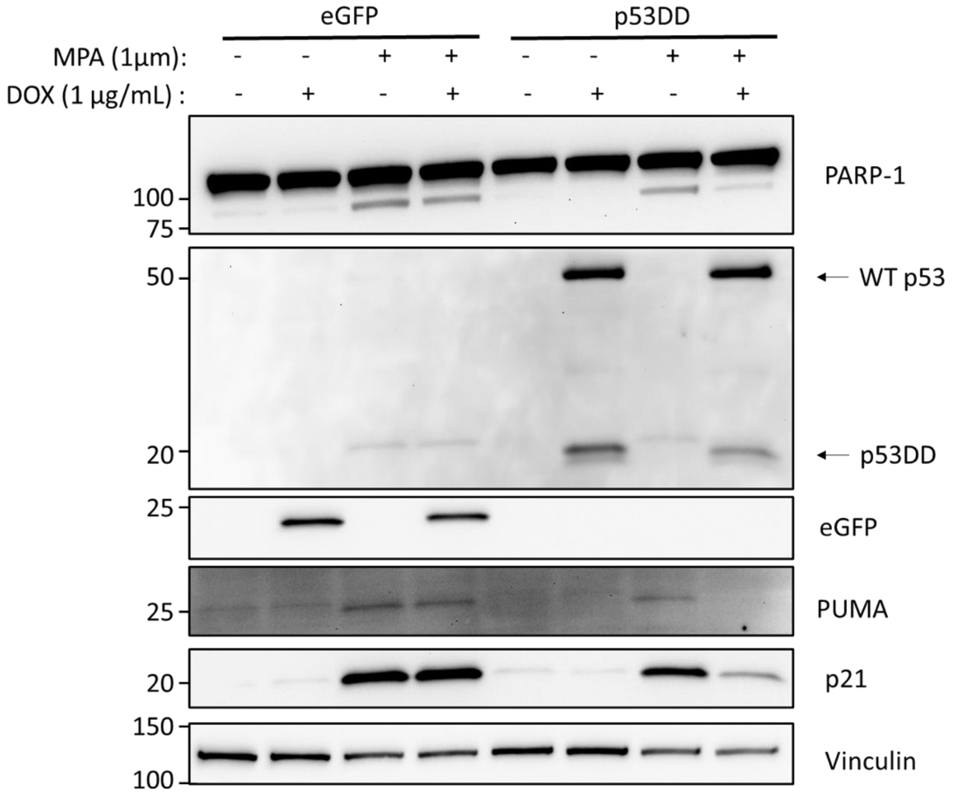

Replicate 3

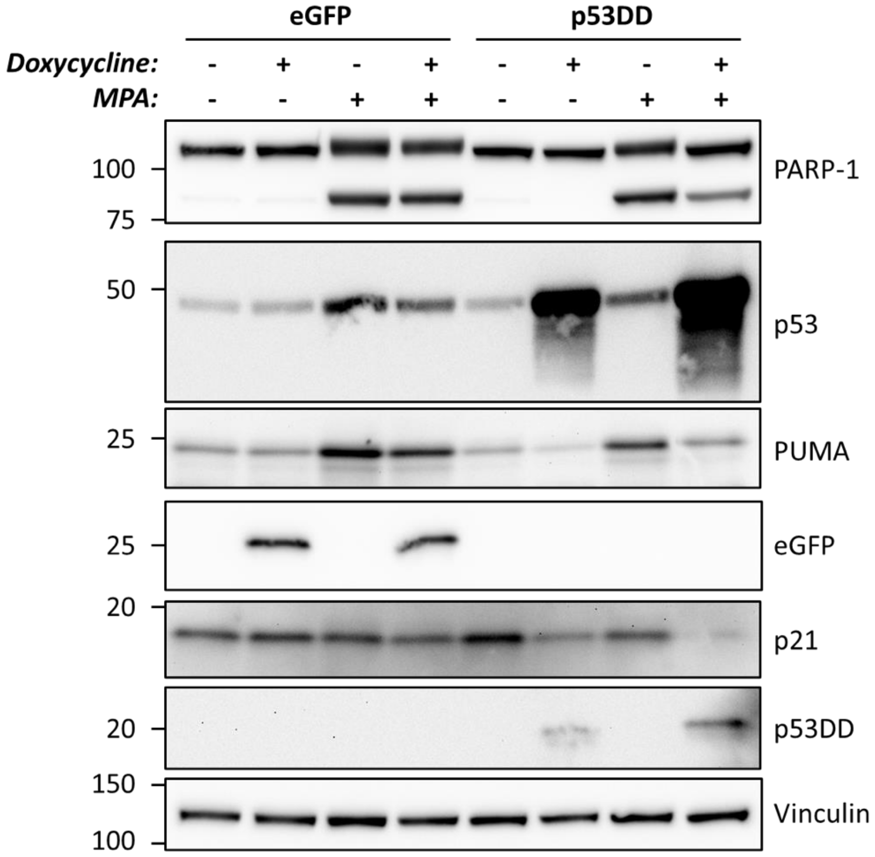

Figure 2B Replicates

Replicate 2

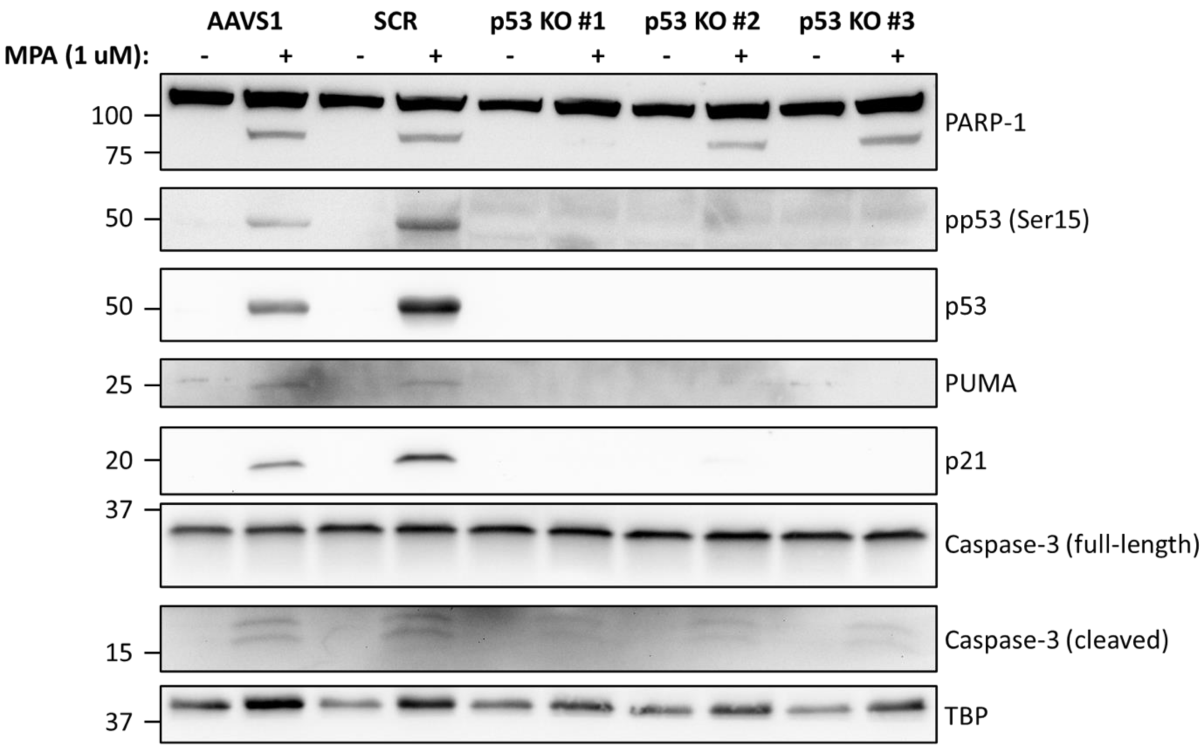

Replicate 3

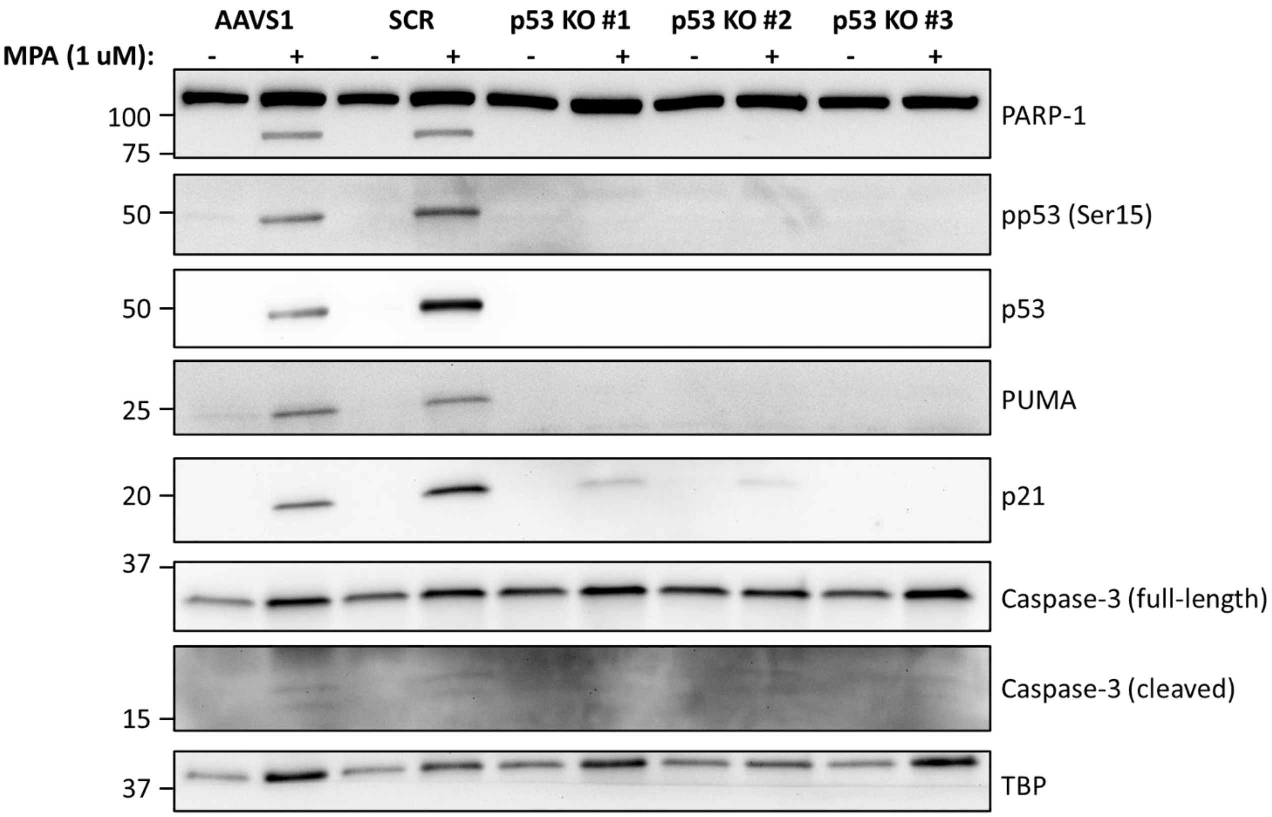

Figure 5A Replicate

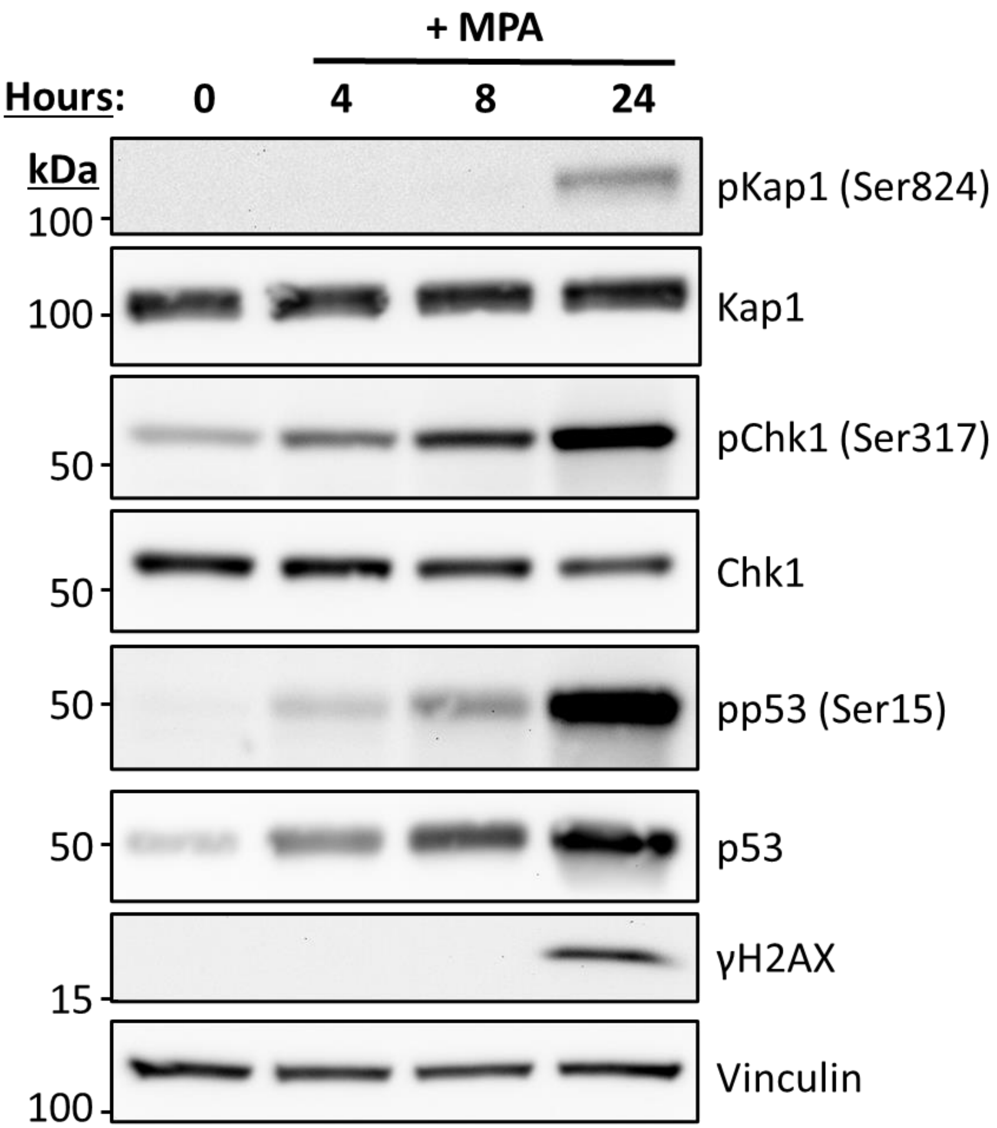

Figure 5B Replicate

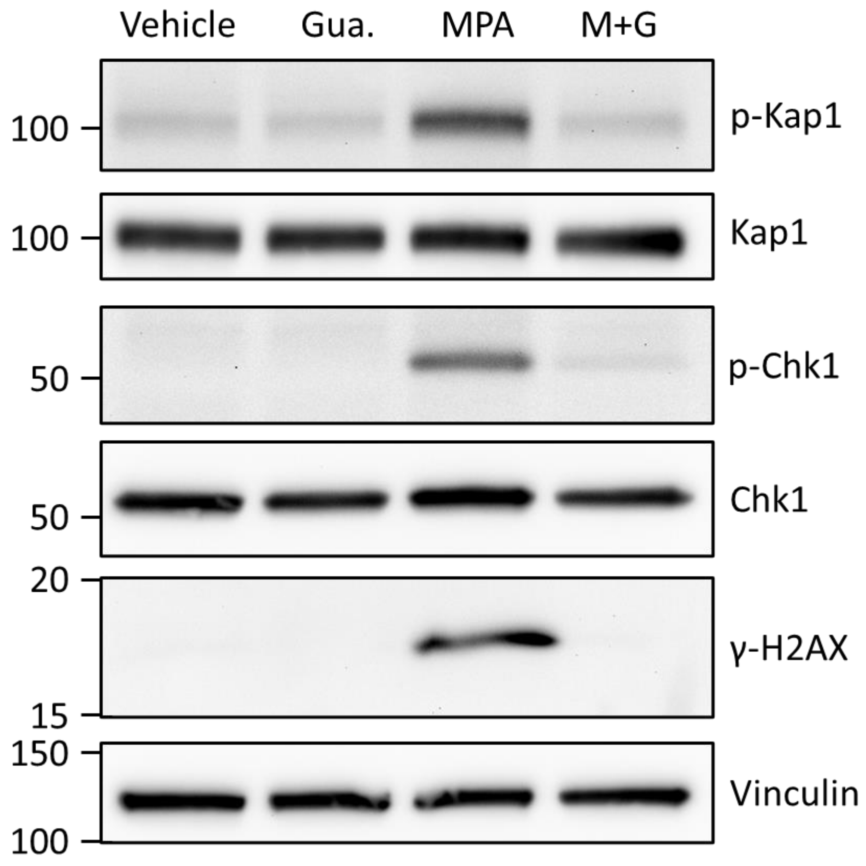

Figure 5C Replicate

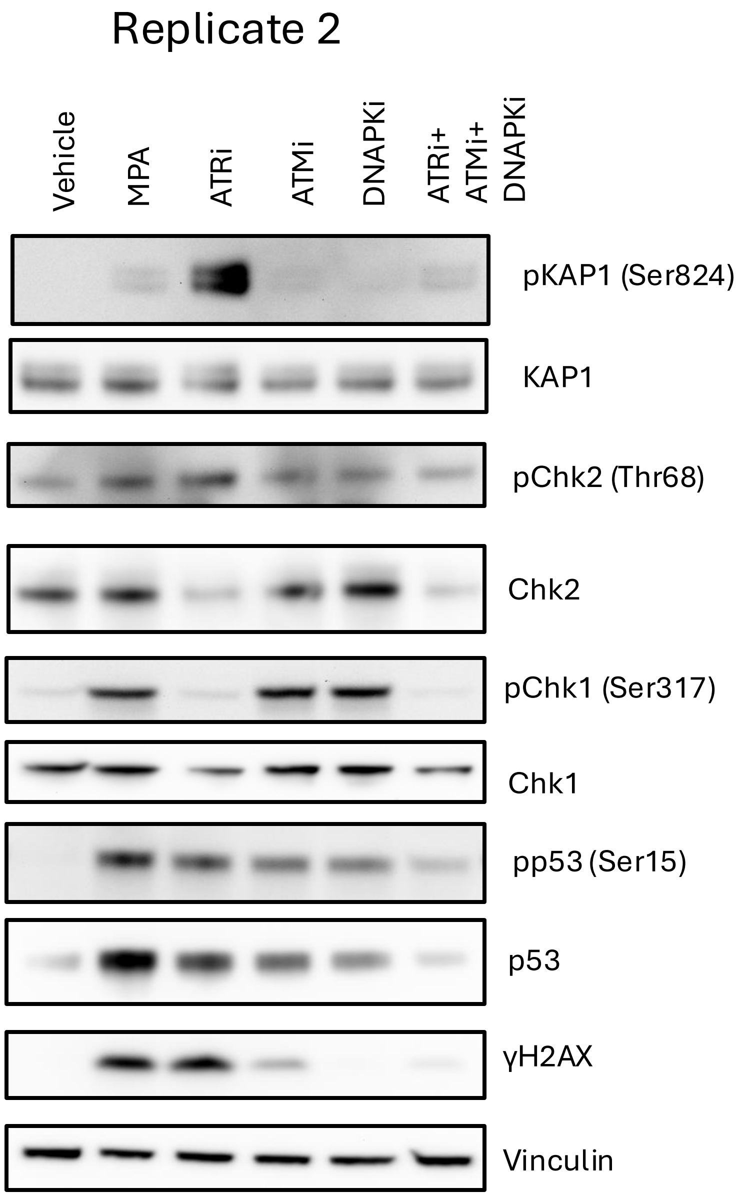

Figure 6A Replicates

Replicate 2

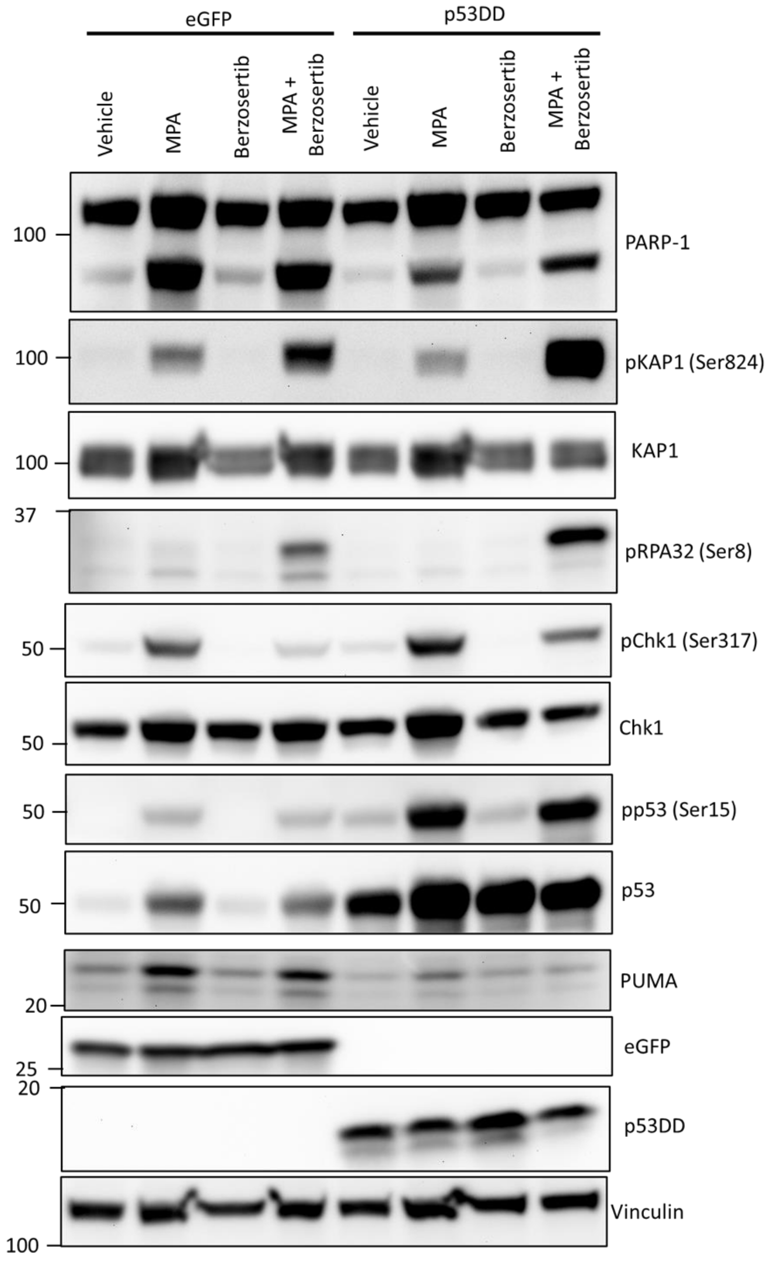

Replicate 3

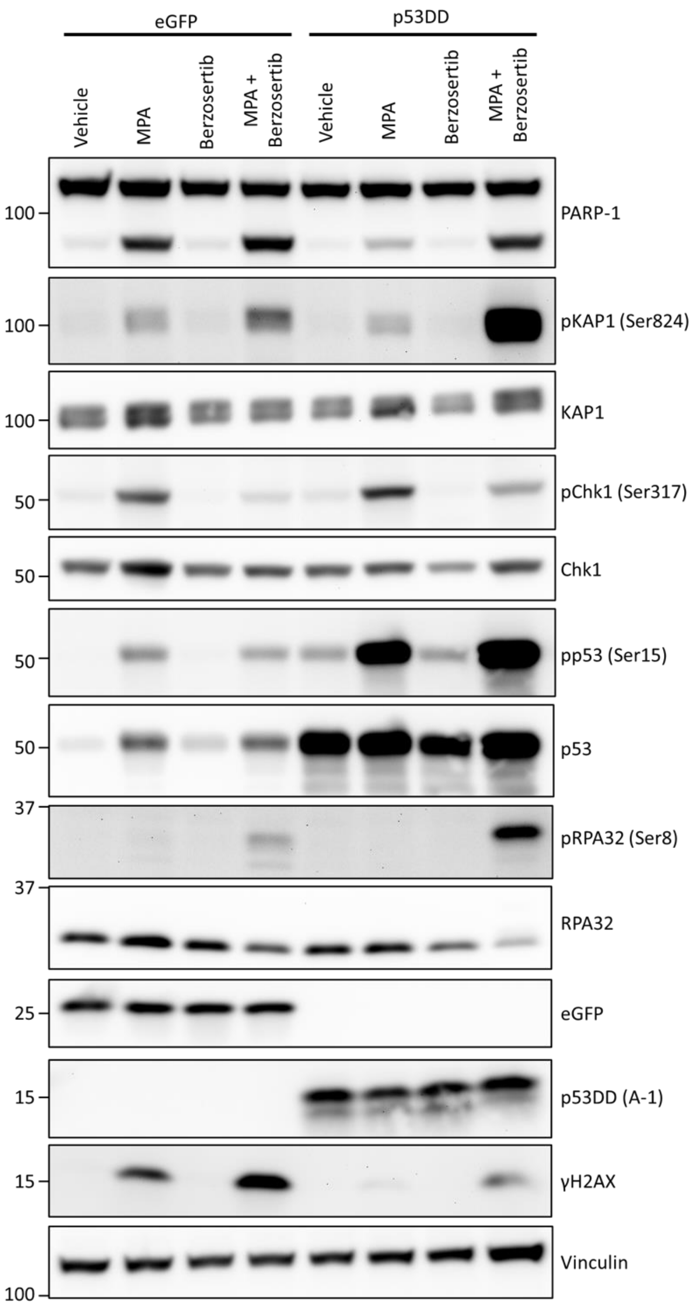

Figure 6B Replicate

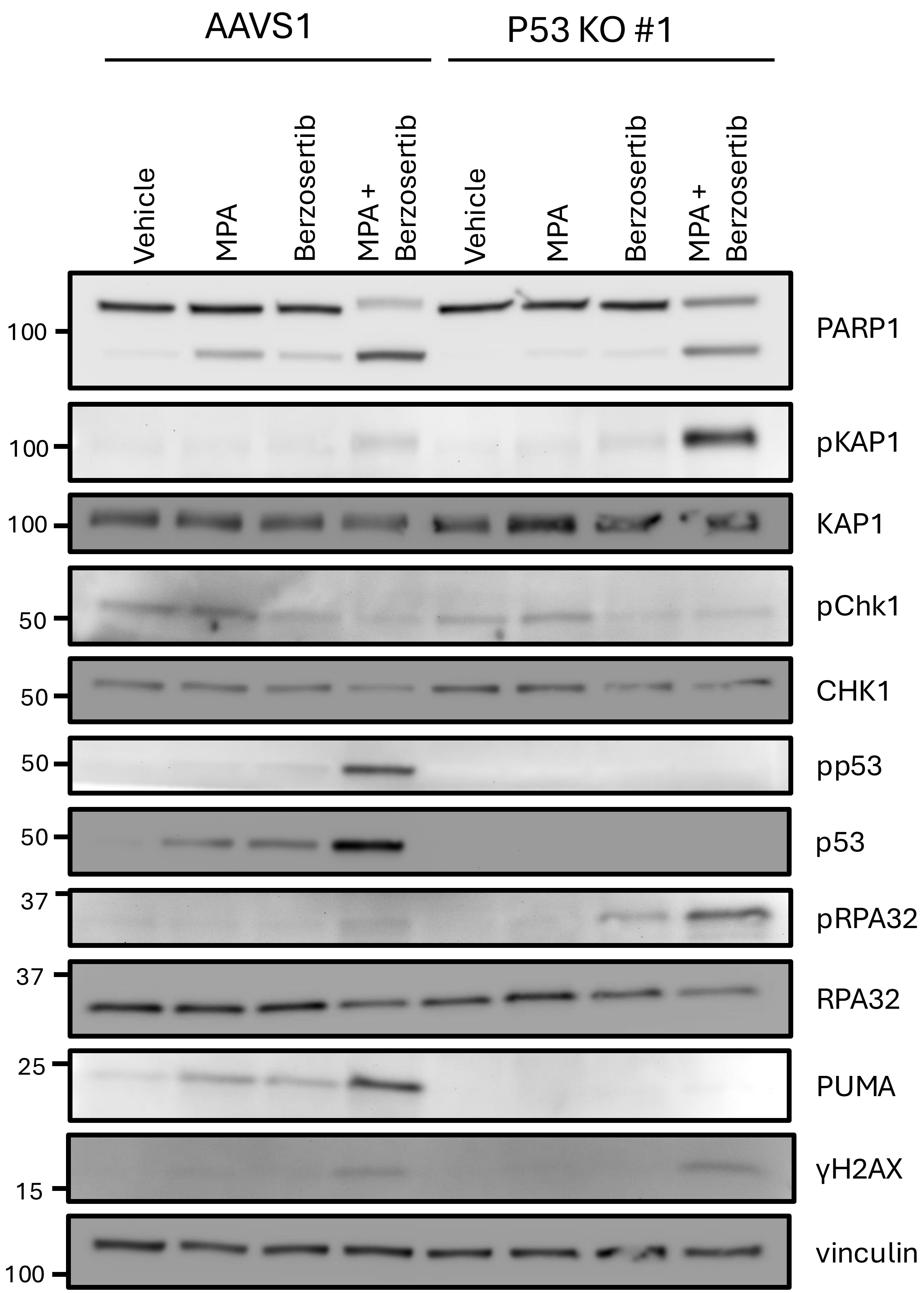

Supplement: Document S2. Appendix containing western blot replicates where available for manuscript figures [file mmc2.pdf]
